# OPN silencing reduces hypoxic pulmonary hypertension via PI3K-AKT-induced protective autophagy

**DOI:** 10.1038/s41598-024-59367-y

**Published:** 2024-04-15

**Authors:** Rui Zhou, Ran Li, Qi Ding, Yuwei Zhang, Hui Yang, Ying Han, Chuanchuan Liu, Jie Liu, Shenglan Wang

**Affiliations:** 1 Qinghai University Medical Department, Xining, 810016 China; 2 Zhengzhou Medical and Health Vocational College, Zhengzhou, 452385 China; 3 Pathology Department of Tianjin Huanghe Hospital, Tianjin, 300110 China; 4 Department of Public Health, School of Medical, Qinghai University, Xining, 810016 China; 5 Key Laboratory of Hydatid Disease, Qinghai University, Xining, 810001 China

**Keywords:** Cardiovascular biology, Macroautophagy

## Abstract

Hypoxic pulmonary hypertension (HPH) is a pulmonary vascular disease primarily characterized by progressive pulmonary vascular remodeling in a hypoxic environment, posing a significant clinical challenge. Leveraging data from the Gene Expression Omnibus (GEO) and human autophagy-specific databases, osteopontin (OPN) emerged as a differentially expressed gene, upregulated in cardiovascular diseases such as pulmonary arterial hypertension (PAH). Despite this association, the precise mechanism by which OPN regulates autophagy in HPH remains unclear, prompting the focus of this study. Through biosignature analysis, we observed significant alterations in the PI3K-AKT signaling pathway in PAH-associated autophagy. Subsequently, we utilized an animal model of OPN^fl/fl^-TAGLN-Cre mice and PASMCs with OPN shRNA to validate these findings. Our results revealed right ventricular hypertrophy and elevated mean pulmonary arterial pressure (mPAP) in hypoxic pulmonary hypertension model mice. Notably, these effects were attenuated in conditionally deleted OPN-knockout mice or OPN-silenced hypoxic PASMCs. Furthermore, hypoxic PASMCs with OPN shRNA exhibited increased autophagy compared to those in hypoxia alone. Consistent findings from in vivo and in vitro experiments indicated that OPN inhibition during hypoxia reduced PI3K expression while increasing LC3B and Beclin1 expression. Similarly, PASMCs exposed to hypoxia and PI3K inhibitors had higher expression levels of LC3B and Beclin1 and suppressed AKT expression. Based on these findings, our study suggests that OPN^fl/fl^-TAGLN-Cre effectively alleviates HPH, potentially through OPN-mediated inhibition of autophagy, thereby promoting PASMCs proliferation via the PI3K-AKT signaling pathway. Consequently, OPN emerges as a novel therapeutic target for HPH.

## Introduction

Hypoxic pulmonary hypertension (HPH) represents a distinct subtype within the broader category subtype of pulmonary arterial hypertension (PAH) and poses a significant burden on patients' quality of life. Characterized by persistent elevation of pressure in the pulmonary arteries, HPH stems from pathological alterations in lung vasculature^1^. These changes lung vasculature irreversible damage to the pulmonary vasculature triggered by oxygen deprivation^2^. In a study conducted in Spiti Valley, India, the prevalence of primary HPH in the local population was 3.23%^3^. HPH is a prevalent and life-threatening condition in highland regions^4^. Current therapeutic approaches for HPH, primarily consisting of long-term oxygen therapy and systemic vasodilators, offer only temporary relief from hypoxic injury to pulmonary vasculature^5^. However, targeted therapeutic strategies specifically addressing pulmonary vasculature lesions in HPH are lacking. Therefore, it is imperative to elucidate the underlying mechanistic pathways to improve survival rates among affected individuals.

Autophagy, a fundamental biological process involving the degradation of internal components within lysosomes such as proteins and mitochondria, has garnered attention for its potential relevance to HPH pathogenesis^6^. Previous studies have identified several aspects of autophagy, including macroautophagy, microautophagy, and molecular chaperone-mediated autophagy, each characterized by distinct cargo delivery mechanisms to lysosomes for degradation^7^. Macroautophagy (hereinafter referred to as autophagy) involves the recognition of cargoes by autophagic vesicles characterized by a double membrane structure. These vesicles encapsulate cargoes and facilitate their binding to lysosomes, where subsequent digestion of their contents occurs^8^. In contrast, microautophagy directly involves the invagination of cargoes by lysosomes for phagocytosis and decomposition^9^. Another variant, molecular chaperone-mediated autophagy relies on the receptor protein LAMP2A expressed on lysosome membranes to selectively recognize cargoes bearing the KFERQ motif. Subsequently, these cargoes traverse specialized channels within the lysosomal membrane, facilitating their entry into the lysosome for degradation^10^. Studies have established a correlation between autophagy and HPH, wherein increased autophagic activity was observed in PASMCs within a rat model aimed at alleviating systolic pressure and attenuating remodeling, thereby impeding the progression of HPH in the pulmonary arteries^11^. This finding suggests that upregulation of autophagy may hold promise in preventing HPH progression. Furthermore, treatment with tanshinone II sodium sulfonate A has been shown to stimulate autophagy in rat lung tissue under hypoxic conditions, mitigating pathogenic alterations in lung tissue^12^. Thus, modulating autophagy presents a potential therapeutic approach for managing HPH.

OPN, also known as secreted phosphoprotein 1 (SSP1), is a member of the matricellular protein family and is classified as a non-structural extracellular matrix protein involved in diverse cellular processes^13^. Upregulation of OPN expression has been reported in hepatocellular carcinoma (HCC), where it promotes the proliferation and migration of HCC cells^14^. Inhibition of OPN leads to the suppression of cancer cell proliferation, as well as decreased regeneration and survival of primary hepatocytes, and cell cycle arrest^15^. Additionally, OPN has been implicated in the modulation of autophagy, where it attenuates fibrosis in atrial fibroblasts by inhibiting autophagy^16^. It also enhances autophagy capacity in human aortic smooth muscle cells, thereby reducing vascular calcification^17^. Despite these insights, the precise mechanism through which OPN regulates HPH remains elusive. Previous studies have demonstrated the efficacy of triptolide in inhibiting vascular remodeling in rats with HPH by targeting the PI3K-AKT signaling pathway^18^. Notably, differential expression of PI3K, rather than AKT, has been observed when comparing patients with PAH (mPAP ≥ 30) to control subjects (mPAP ≤ 20)^19^. Abnormal activation of PI3K has been implicated in the context of HPH/PAH. However, the involvement of OPN in autophagy in HPH remains insufficiently investigated.

In this study, we employed bioinformatics techniques to identify OPN as a common gene intersecting PAH and autophagy. Subsequently, we identified the top ten genes common among differentially expressed genes (DEGs), autophagy-related genes (ARGs), and differentially modular genes (DMGs), considering them as hub genes. KEGG analysis of these genes identified the PI3K-AKT pathway as one of the prominent pathways. To investigate the role of OPN in HPH, we generated OPN^fl/fl^-Cre CB57 mice. This study aimed to examine the impact of OPN on HPH progression and explore its potential regulatory effects on autophagy and the proliferation of PASMCs through the PI3K signaling pathway. The objective of this research was to elucidate the influence of OPN on autophagy and its involvement in pathological changes in pulmonary artery smooth muscle in the HPH model via the PI3K signaling pathway. These findings provide potential avenues for the development of therapeutic interventions for HPH.

## Methods

### Data collection and processing

**Figure 1:**
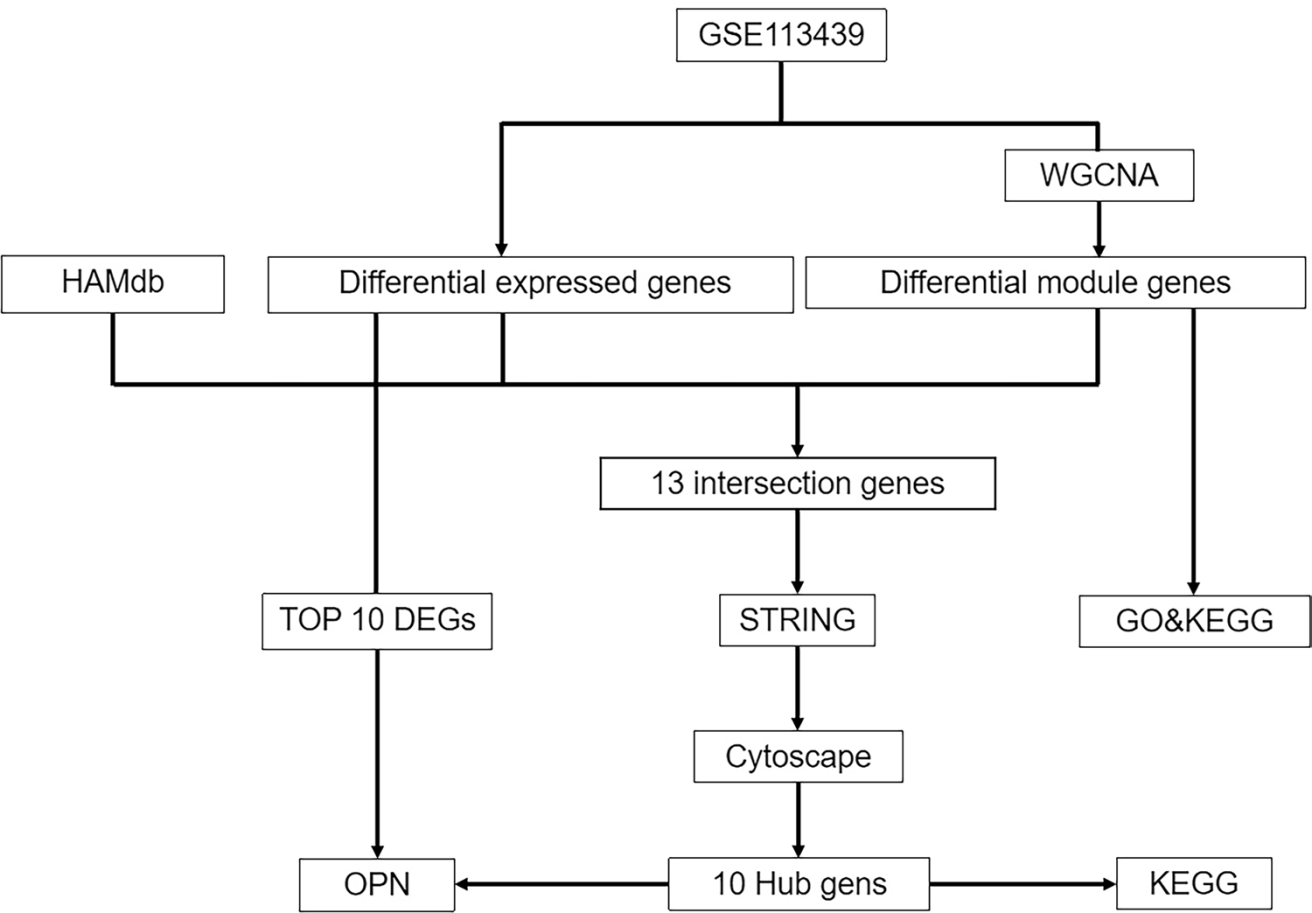
Bioinformatics data analysis process.

Gene expression profiles of GSE113439^20^ were downloaded from a publicly available gene/gene microarray gene fragment database, the Gene Expression Omnibus (GEO) database (http://www.ncbi.nlm.nih.gov/geo (accessed December 3, 2022)).GSE113439 is based on the GPL6244 (Affymetrix Human Gene 1.0 ST Array) platform and contained 15 cases of PAH (the PAH group consisted of 6 patients with idiopathic PAH, 4 patients with PAH secondary to connective tissue disease, 4 patients with PAH secondary to congenital heart disease and 1 patient with chronic thromboembolic pulmonary hypertension and 11 controls (normal lung tissues obtained from flank lung cancer resections)) for transcriptomic information. The GPL file with matrix file for GSE113439 and autophagy-related genes (ARGs) data were downloaded from the Human Autophagy Database (http://hamdb.scbdd.com/home/index/ (accessed December 17, 2022)) for this study to facilitate subsequent analysis. Figure 1 showed the flowchart of bioinformatic data analysis in this study.

### Analysis of differentially expressed genes (DEGs)

The DEGs were analyzed using the "limma" package^21^ in the R software (version 4.2.2.) Specifically, this study used the Imfit function to find multiple linear regressions on the dataset. Then, we used the eBays function to compute the regulation t-statistic, the regulation F-statistic, and the log odds of differential expression by empirical Bayesian adjustment of the standard error to the common value. Finally, we obtained the significance of the difference for each gene. We set the fold change to log Fold Change > 1 and adjusted the p-value to set it to less than 0.01 to screen for target genes. Differential genes were then visualized using the "ggplot2" and "heatmap" R packages for volcano maps of all differential genes and heatmaps of the top 50 differentially up-and-down-regulated genes.

### Weighted gene co-expression network analysis (WGCNA)

WGCNA is an algorithm that evaluates the relationships between measured transcripts, identifies clinically relevant co-expressed gene modules, and explores key genes in disease pathways from a systems biology perspective^22^. The “WGCNA” R package was used to construct the PAH correlation module. To implement the scale-free network, the "pickSoftThreshold" function in the package was used to determine the optimal soft-threshold power β for increasing the expression similarity and calculating the neighboring relationships. Next, the gene correlation matrix was transformed into a neighboring matrix, which was further converted into an unsigned topological overlap matrix (TOM). According to the TOM, average chained hierarchical clustering was used to obtain gene clusters and construct a dendrogram. A minimum module size of 30 genes was used to identify gene modules using a dynamic tree-cutting algorithm (deep Split = 2); genes with similar expression patterns were assigned to the same module. Module-characterized genes (MEs) were calculated as the first principal component of the expression profile in each module. Modules were then clustered and merged based on ME differences (merge Cut Height = 0.25). The correlation between MEs and clinical characteristics of PAH patients was calculated using the Pearson correlation coefficient. Then, the two modules with the highest coefficients were targeted and the genes within the two modules were extracted for further analysis.

### Functional enrichment analysis

Genes enriched in blue and turquoise modules with gene significance greater than 0.5 in WGCNA were analyzed by Gene Ontology (GO) annotation and Kyoto Encyclopedia of Genes and Genomes (KEGG) pathway enrichment analysis^23–25^ using the "cluster Profiler" R (version 4.2.2) package^26^, including biological process (BP), cellular component (CC), molecular function (MF) and KEGG pathway enrichment analysis visualized by the "ggplot2" R package. BP, CC, MF, and KEGG pathway enrichment analyses were included and visualized by the "ggplot2" R package. The P-value was set to 0.05 as the critical value.

### Identification of differential expressed module autophagy-related genes (DEMARGs) and construction of protein–protein interaction networks

We used the "Veen" R (version 4.2.2) package to identify DEGs, differential module genes, and ARGs co-expressed in DEMARGs. Subsequently, protein–protein interaction (PPI) between DEMARGs was analyzed and visualized by the CYTOSCAPE (version 3.9.1) software using the Searching for Gene Interactions Search Tool (STRING) database (https://cn.string-db.org (accessed December 18, 2022)) to analyze PPI between DEMARGs and visualize them by CYTOSCAPE (version 3.9.1) software^27^. The DEMARGs were ranked using four algorithms, MCC, MNC, Degree, and EPC^28^ in the CytoHubba plugin to obtain the top ten Hub genes, and the Hub genes were analyzed for KEGG enrichment.

### GeneMANIA: gene pathways and interactions of Hub genes

GeneMANIA (http://www.genemania.org (accessed February 18, 2023)) provides a flexible, user-friendly analysis web interface for generating hypotheses based on gene function, analyzing gene lists, and prioritizing genes for functional analysis^29^. GeneMANIA was used to construct the gene–gene interaction network of Hub genes from physical interactions, co-expression, prediction, co-localization, and genetic interactions, and to evaluate their functions.

### Animals

C57BL/6J (wide-type) mice were used as background. Transgelin protein encoded by the transgelin (TAGLN) gene is involved in regulating the formation and maintenance of the cytoskeleton, influencing cell contraction, morphology, and migration, and is an early marker of smooth muscle differentiation. To construct the targeting vector, BAC clone RP24-190A7 was used as a template to generate homologous Bo and cKO regions by PCR. Cas9 protein, sgRNA and targeting vector were co-injected into mouse fertilized eggs to generate F0 mice. F0-positive mice were mated with WT (TAGLN-Cre-containing) mice to obtain F1 generation OPN^flox/+^, TAGLN-Cre heterozygous mice that can be stably inherited. F1 generation OPN^flox/−^, TAGLN-Cre mice were self-crossed with mice of the same genotype to obtain pure and OPN^flox/flox^, TAGLN-Cre mice as vascular smooth muscle-specific knockout mice for experiments. Controls were littermate control OPN^flox/flox^ mice (10–12 weeks) weighing 22 to 25 g for this study. All mice were purchased from Cyagen Biological Research Center (Taicang, China) (license SCXK (Su) 2018–0003). They were housed in a temperature-controlled environment at 22 ± 2 °C with a relative humidity of 45–55% and were fed ad libitum with a standard laboratory diet and tap water for 1 week before the experiment. The animals were randomly divided into 4 groups: (1) normoxic control using OPN^flox/flox^ control mice (n = 7, hereafter referred to as normoxia group); (2) normoxic using OPN^flox/flox^, TAGLN-Cre mice (n = 7, hereafter referred to as normoxia + OPN^fl/fl^-TAGLN-Cre group); (3) hypoxic environment using OPN^flox/flox^ control mice (n = 7, hereafter referred to as hypoxia group); (4) using OPN^flox/flox^, TAGLN-Cre mice in a hypoxic environment (n = 7, hereafter referred to as hypoxia + OPN^fl/fl^-TAGLN-Cre group). 3 and 4 groups were housed in a DYC3000 low-pressure hypoxic (10.6% oxygen content relative to sea level) chamber (Fenglei, Guizhou, China) at a simulated altitude of 5000 m for 28 days, and all animals were kept in a 12-h light-12-h dark cycle at 22 ± 2 °C, with food provided ad libitum, and bedding changed every 3 days.

### Mean pulmonary artery pressure (mPAP) measurement in mice

A 2% sodium pentobarbital anesthetic was injected into the peritoneal cavity of mice according to their body weight. When the mice were in deep anesthesia, the mice were placed supine and fixed on the mouse platform, and a cut was made in the middle of the anterior neck to locate and separate the right measured external jugular vein, and a polyethylene microcatheter was heparinized and inserted from the right external jugular vein to the pulmonary artery. The other end was connected to a BL-420S biopressure transducer (Ed Instruments, Shanghai, China), and the pulmonary artery pressure profile was recorded for 5 min.

### Measurement of right ventricular hypertrophy index (RVHI) in mice

Immediately after the dislocation and execution of mice under anesthesia, heart, and lung tissues were removed, the surface was cleaned of blood with saline, and the whole heart was weighed, the right ventricle (RV) was cut out and the RV was weighed, followed by isolation of the left ventricle (LV) and the interventricular septum (S), and weighing the LV and the S. The right ventricle was then removed from the right ventricle and the septum was removed from the right ventricle. The result of calculating the Fulton index (RV/LV + S) represented the right ventricular hypertrophy index (RVHI).

### Transmission electron microscopy

Pulmonary arteries and PASMCs from all groups were fixed by adding 3% glutaraldehyde fixative at 4 °C for 24 h, and 1% osmium tetroxide was added and fixed for another 2 h. The fixed samples were dehydrated stepwise by immersing them in acetone and then embedded in epoxy resin after completion of dehydration. The samples were prepared into 50 nm sections, and the sections were stained with lead citrate and placed under a JEM-1400FLASH transmission electron microscope (JEOL, Tokyo, Japan) for observation and image acquisition.

### Culturing of primary PASMCs

Ten 6-week-old SD rats (Certificate of Conformity No. 110322220100347884) purchased from Beijing Huafu Biotechnology Company were euthanized by cervical dislocation after being anesthetized by intraperitoneal injection of 2% sodium pentobarbital and were sterilized in 75% ethanol for 3 min. The heart and lung tissues were taken out by opening the thoracic cavity in an ultra-clean bench and placed in Petri dishes containing pre-cooled sterile 1% PBS at 4 °C (Solepol, Beijing, China)) in a Petri dish. The heart was removed, and the lung tissue was washed with PBS, and the lung tissue was fixed in a Petri dish containing floatation. Secondary and tertiary pulmonary arteries were isolated step by step down the main pulmonary artery trunk. They were transferred to a new petri dish for cleaning and then the small pulmonary arteries were cut longitudinally, the endothelial cells were gently scraped with a scalpel, and the outer and middle membranes were separated with ophthalmic forceps. The middle smooth muscle tissue was cut into 1 mm^3^-sized tissue blocks, which were then transferred to 15 mL centrifuge tubes containing 1–2 mL of 0.2% type II collagenase (Solebol, Beijing, China), and the centrifuge tubes were placed in a water bath at 37 °C for digestion for about 1 h. Digestion was terminated when the tissue blocks became flocculent. After digestion, the cells were resuspended in high glucose DMEM medium (Procell, Wuhan, China) containing 20% FBS (Gibco, California, USA), and the resuspension solution was added into the culture flasks and placed in a humidified incubator at 37 with 5% CO_2_ (Thermo HERAcell150i, Thermo Fisher Scientific, America) for culture. When the cells grew to about 70% confluence, the cells were purified by differential wall affixation. Generation 3–5 cells were used for subsequent experimental studies. In addition, the cells were classified into normoxia, hypoxia, hypoxia + OPN shRNA EV, hypoxia + OPN shRNA, and hypoxia + LY294002 (PI3K inhibitor) groups. Normoxic PASMCs were placed in an ambient incubator (Thermo HERAcell 150i, ThermoFisher, USA) with 5% CO_2_ and 20% O_2_ for 48 h, and hypoxic PASMCs were placed in an ambient incubator (CB53, BINDER, Germany) with 5% CO_2_ and 1% O_2_ for 48 h and then used for subsequent cell experiments.

### Immunocytochemical assay

Logarithmic growth phase PASMCs were digested with 0.2% trypsin (Solebol, Beijing, China), and 1 × 10^4^ cells were inoculated into 6-well cell culture plates. After cell attachment, the original medium was discarded, the cells were washed twice with PBS, and 4% paraformaldehyde was added to fix the cells at room temperature for 15 min. The cells were subsequently processed according to the steps of the two-step immunohistochemistry kit (Elabscience, Wuhan, China). Used to identify PASMCs, brown cells represent PASMCs.

### Reverse transcription-polymerase chain reaction (RT-PCR)

Total RNA from lung tissues was extracted using the Total RNA Extraction Kit (TIANGEN, Beijing, China) according to the manufacturer's instructions. cDNA was synthesized using the Reverse Transcription Reagent (TIANGEN, Beijing, China). cDNA was extracted from lung tissues using SuperReal PreMix Color (SYBR Green) (TIANGEN, Beijing, China) to determine the gene expression levels in an ABI PRISM 7500 sequence detection system (Applied Biosystems, Foster City, USA). Transcript expression levels were normalized to endogenous β-actin expression levels. All primer sequences were shown in Supplementary Table 1.

### Cellular lentiviral transfection and culture

OPN interference sequences were designed, forward ‘GATGTCCCTFACGGCCGAGGT’, reverse ‘ACCTCGGCCGTCAGGGGACATC’. Logarithmic growth phase PASMCs were inoculated in 25 mm^2^ culture flasks according to the instructions of the company from which they were purchased (Cyagen, California, America), and the cells were transfected with OPN interference sequences when the cells had grown to 30–40%. The virus was first lysed in a disease bath, polybrene was added to the virus-containing medium, and the viral solution was allowed to cover the surface of all cells overnight, and the virus-containing medium was removed the day after transfection to add fresh complete medium. After the virus-containing cells stably expressed specific green fluorescence, the cells were collected for subsequent experiments. The PASMCs were categorized into the hypoxia + OPN shRNA EV group plus containing OPN empty virus; and hypoxia + OPN shRNA group plus containing OPN interfering with lentivirus.

### Western blotting (WB)

Lung tissues and cells of each group were collected, and the supernatant was collected after lysis on ice by adding the appropriate amount of RIPA lysate. Protein concentration was detected by the BCA (No. 23227, Thermo Fisher Scientific) method. Polyacrylamide gel electrophoresis (SDS-PAGE) was performed with 30 µg of protein per well, and the target proteins were transfected onto a PVDF membrane, which was closed with 5% skimmed milk powder at room temperature for 1 h. To reduce the number of primary antibodies used, we cut the membranes to the appropriate size based on the marker corresponding to the molecular weight of the protein before incubating the membranes with the following primary antibody. The membranes were incubated with LC3B (1:1000, No. ab192890, Abcam), Beclin1 (1:2000, No. ab207612, Abcam), OPN (1:1000, No. ab63856, Abcam), PI3K (1:2000, No. ab191606, Abcam), AKT (1:1000, No. ab8805, Abcam), β-actin (1:5000, No. AC026, ABclonal) antibodies were incubated overnight at 4 °C in the refrigerator. The membrane was washed three times with TBST on the following day and then added with anti-mouse HRP secondary antibody (1:10,000, No. AS003, ABclonal) or anti-rabbit HRP secondary antibody (1:10,000, No. AS014, ABclonal) and incubated at room temperature for 1 h. After washing, the membrane was washed with ultrasensitive luminescent solution (No. 1856189, Thermo Fisher Scientific, America) in a gel imager to develop and save the images and analyze the gray value of the bands with ImageJ software (version 1.53t) to calculate the relative protein expression with β-actin as an internal reference.

### 5-ethynyl-2'-deoxyuridine (EdU) staining

EdU staining was performed using the BeyoClick™ EdU Cell Proliferation Detection Kit (BeyoClick, Nanjing, China). Cells were inoculated in 6-well plates and stained with 50 μM EdU for 2 h. Subsequently, cells were washed twice with PBS, fixed with 50 μL of fixative (PBS + 4% polyoxymethylene), and incubated for 30 min. Finally, cells were discolored with 100 μL of permeabilization agent (PBS + 0.5% TritonX-100) for 2–3 times (each rinse for 10 min). the nuclei were stained with DAPI staining of nuclei was performed for 10 min. Cell staining results were observed with an inverted fluorescence microscope and EdU positively stained cells were counted using ImageJ software (version 1.53t).

### Flow cytometry

PASMCs were inoculated in 6-well plates at 8 × 10^4^ cells/well. PASMCs were grown in normoxia, hypoxia, hypoxia empty virus, hypoxia OPN shRNA, and hypoxia LY294002 culture environments. The normal untreated normoxia group served as a control. Cells were treated with a cell cycle assay kit (cell cycle assay kit, E-CK-A351, Wuhan, China). 48 h later, PASMCs were cultured in a conditioned medium collected in 1.5 mL centrifuge tubes, and the cells were washed with precooled PBS, and fixed in pre-cooled anhydrous ethanol at − 20 °C for 1 h. After washing again with PBS, 100 μL of RNase A Regent in a water bath at 37 °C for 0.5 h. After washing again with PBS, 25 μL of PI Regent was added, gently mixed, resuspended, and incubated for 30 min at 37 °C, protected from light, before being detected by flow cytometry to detect changes in the cell cycle. The data were further analyzed using FlowJo software (version 10.8.1).

### Statistical analysis

Statistical analyses were performed using R software (version 4.2.2) or GraphPad Prism (version 9). All data are expressed as mean ± standard deviation (SD) and all experiments were repeated at least 3 times. Significant differences were determined using Bonferroni’s multiple comparison test with a one-way analysis of variance (ANOVA) between the control and other groups. *p* < 0.05 was considered statistically significant.

### Delaration of ethis

This study conformed to the stipulations set forth in the ARRIVE guidelines for experimental animals research. The protocols governing animal care and experimental utilization were rigorously aligned with the Chinese Guidelines for the Care and Use of Laboratory Animals. The animal experiments obtained approval from the Ethics Committee of Qinghai University School of Medicine.

## ResuIts

### DEGs analysis in PAH patients and normal individuals

**Figure 2:**
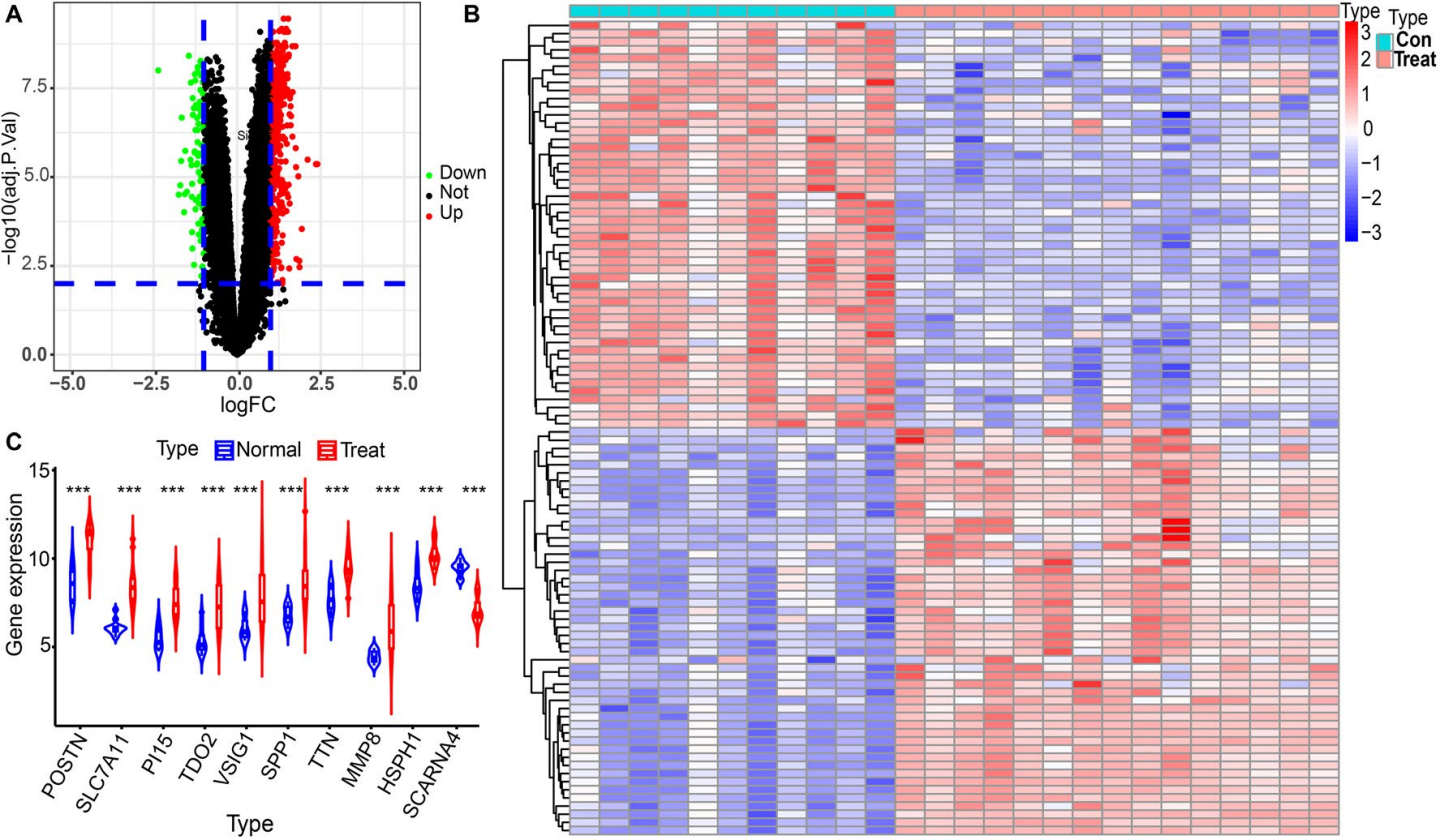
An analysis of the differentially expressed genes. (**A**) Volcano diagram of PAH (GSE113439) data, where green scatters indicated down-regulated genes and red scatters indicated up-regulated genes. (**B**) A heat map of PAH (GSE113439) data, with red showing high expression and blue indicating low expression. (**C**) Violin diagram of the top 10 differentially expressed genes in GSE113439 data. Asterisks indicate statistically significant differences. ***p < 0.001.

Gene expression profiles from the GSE113439 dataset, comprising 15 samples of PAH and 11 samples of normal lung tissue were obtained from GEO. The profiles were examined to identify differentially expressed genes (DEGs) using a threshold of adjusted p-value < 0.01 and |log fold change| > 1. This analysis revealed a total of 555 DEGs, which were further investigated. A volcano plot was generated, illustrating 468 upregulated genes and 87 downregulated genes (Fig. 2A). Additionally, a heatmap was constructed to visually represent bivariate hierarchical clustering results for the top 100 DEGs, including both up-regulated and down-regulated genes (Fig. 2B). The fo Among the 10 genes exhibiting differential expression, POSTN, SLC7A11, PI15, TDO2, VSIG1, SPP1, TTN, MMP8, and HSPH1 were upregulated, while SCARNA4 were downregulated (Fig. 2C). Notably, SPP1 demonstrated significant differential expression in PAH and was among the top 10 genes with differential expression.

### WGCNA and identification of key modules

**Figure 3:**
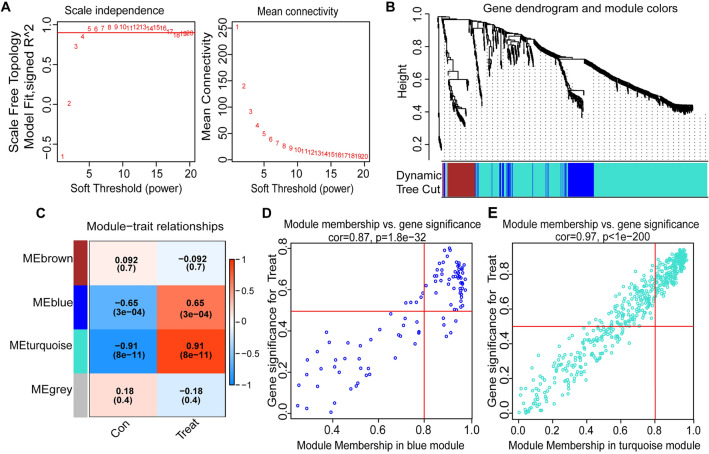
WGCNA of GSE113439 data. (**A**) Scale-free fit index (on the left) and average connectivity (on the right) for evaluating various soft threshold powers. (**B**) The map of differentially expressed genes based on the topological overlap matrix. (**C**) Heatmap displaying the relationship between modules and sample attributes. (**D**,**E**) Scatter diagrams of module genes in modules colored blue and turquoise.

A gene co-expression network was constructed using the GSE113439 dataset and the WGCNA algorithm, with the PAH-related genes as a basis. Using a β value of 4, a scale-free network was generated (Fig. 3A). Subsequently, dynamic hybrid cuts were applied to create a hierarchical clustering tree, resulting in the formation of gene modules. Within the hierarchical tree divisions, several genes exhibited similar patterns, with each gene represented as a leaf (Fig. 3B). Upon analyzing the correlations between modules and traits, a significant association was observed between the MEturquoise and MEblue modules in PAH (Fig. 3C). The turquoise module (correlation coefficient = 0.97, *p* < 1e−200) and blue module (correlation coefficient = 0.87, *p* = 1.8e−32) showed a significant and positive correlation between gene significance and module membership (Fig. 3D,E). Therefore, these two modules were identified as potential sources of differentially expressed module genes (DMGs).

### KEGG and GO enrichment analysis of DMGs

**Figure 4:**
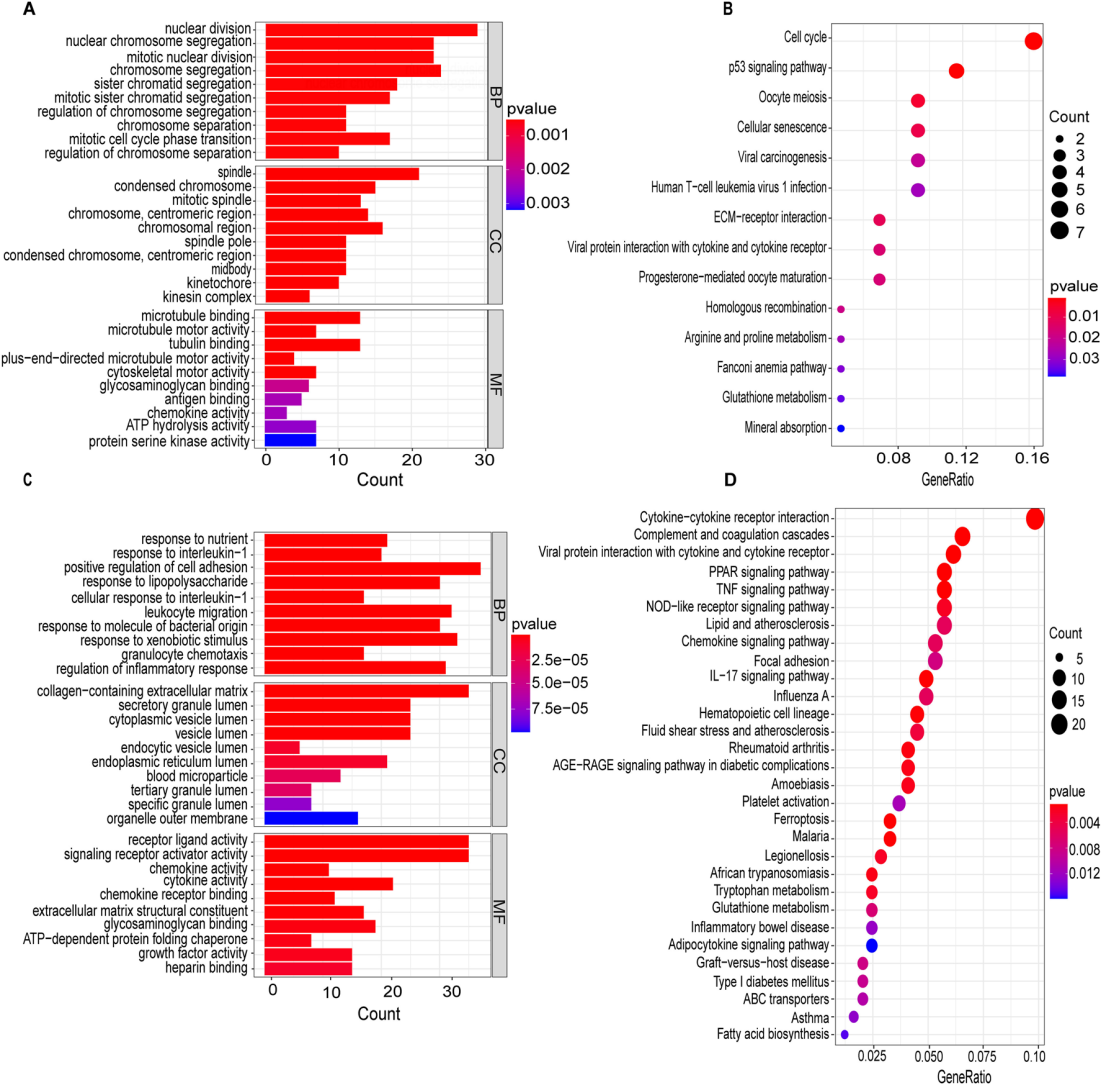
Functional enrichment analysis of blue and green pine module genes using GO and KEGG. (**A**,**C**) Top 10 GO terms in the blue module and turquoise module genes in cellular components, molecular functions, and biological processes, respectively. (**B**,**D**) Blue module and turquoise module genes enriched in the KEGG pathway with *P* < 0.05. Warmer colors indicated higher statistical significance.

To comprehensively understand the biological functions of distinct module genes in PAH, we conducted a GO analysis. This analysis identified 102 genes within the blue module, participating in diverse biological processes including nuclear division, chromosome segregation, and mitotic karyokinesis. These genes were notably enriched in cellular structures such as the spindle, condensed chromosome, and mitotic spindle, and were involved in various cellular functions related to microtubules, including microtubule binding, microtubule motility, and microtubule protein binding (Fig. 4A). Additionally, KEGG analysis revealed that genes within the blue module were associated with several biological processes, such as the cell cycle, P53 signaling pathway, and oocyte meiosis (Fig. 4B). In the turquoise module, GO analysis identified 484 genes implicated in promoting nutritional response, pyridine nucleotide metabolism, and cell adhesion in biological processes. Furthermore, these genes were linked to cellular components including the extracellular matrix containing collagen, the lumen of secretory granules, and cytoplasmic vesicles. Functionally, the turquoise module genes were implicated in many biological functions, including receptor-ligand action, signaling receptor activation, and chemokine activity (Fig. 4C). KEGG analysis of the turquoise module genes revealed significant associations with various biological processes such as cytokine-cytokine receptor interactions, complement and coagulation cascades, as well as viral protein-cytokine and cytokine receptor interactions (Fig. 4D).

### Identification of hub genes

**Figure 5:**
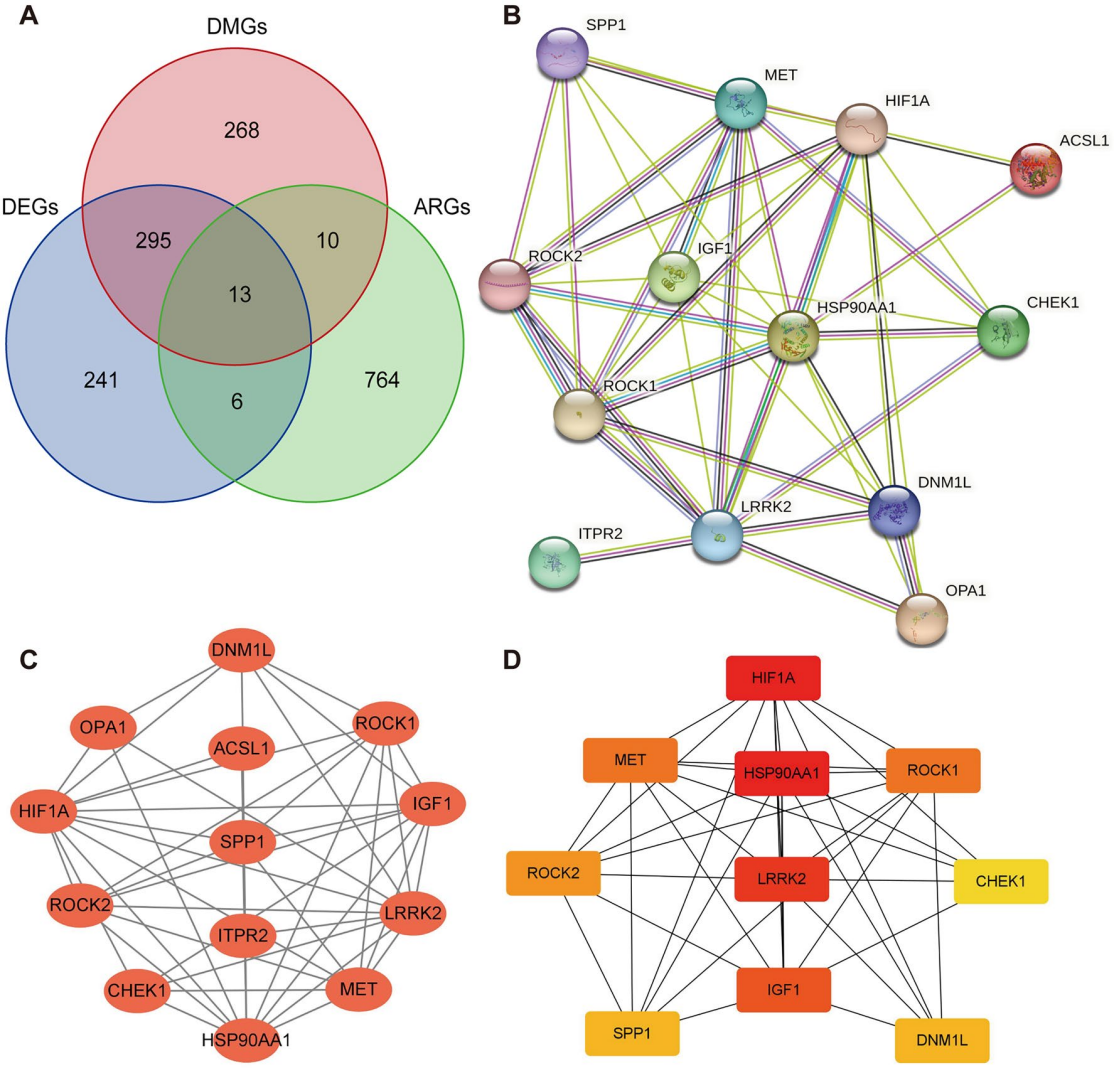
Hub gene PPI construction. (**A**) Genes where ARGs, DEGs, and DMGs intersect. (**B**) STRING-based PPI analysis of DEMARGs. (**C**) Visualization of the PPI of DEMARG using Cytoscape. (**D**) Identification of 10 Hub genes in DEMARGs through MCC, MNC, Degree, and EPC algorithms in CytoHubbs.

A total of 793 autophagy-related genes (ARGs) were obtained from a specialized human database dedicated to autophagy. Subsequently, a Venn diagram analysis (Fig. 5A) was performed on the ARGs, DEGs, and DMGs, resulting in the identification of 13 differential expressed module autophagy-related genes (DEMARGs). To potential protein–protein interactions, the DEMARGs were analyzed using the STRING database, yielding a protein–protein interaction (PPI) network consisting of 13 nodes and 44 edges (Fig. 5B). The composite scores for these interactions ranged from 0.163 and 0.999. The PPI was further visualized using Cytoscape software (Fig. 5C). Additionally, the CytoHubba software plug-in was used to identify the top 10 hub genes, which were determined to be HSP90AA1, HIF1A, LRRK2, IGF1, MET, ROCK1, ROCK2, DNM1L, SPP1, and CHEK (Fig. 5D).

### GenMANIA and KEGG enrichment analysis of the Hub genes

**Figure 6:**
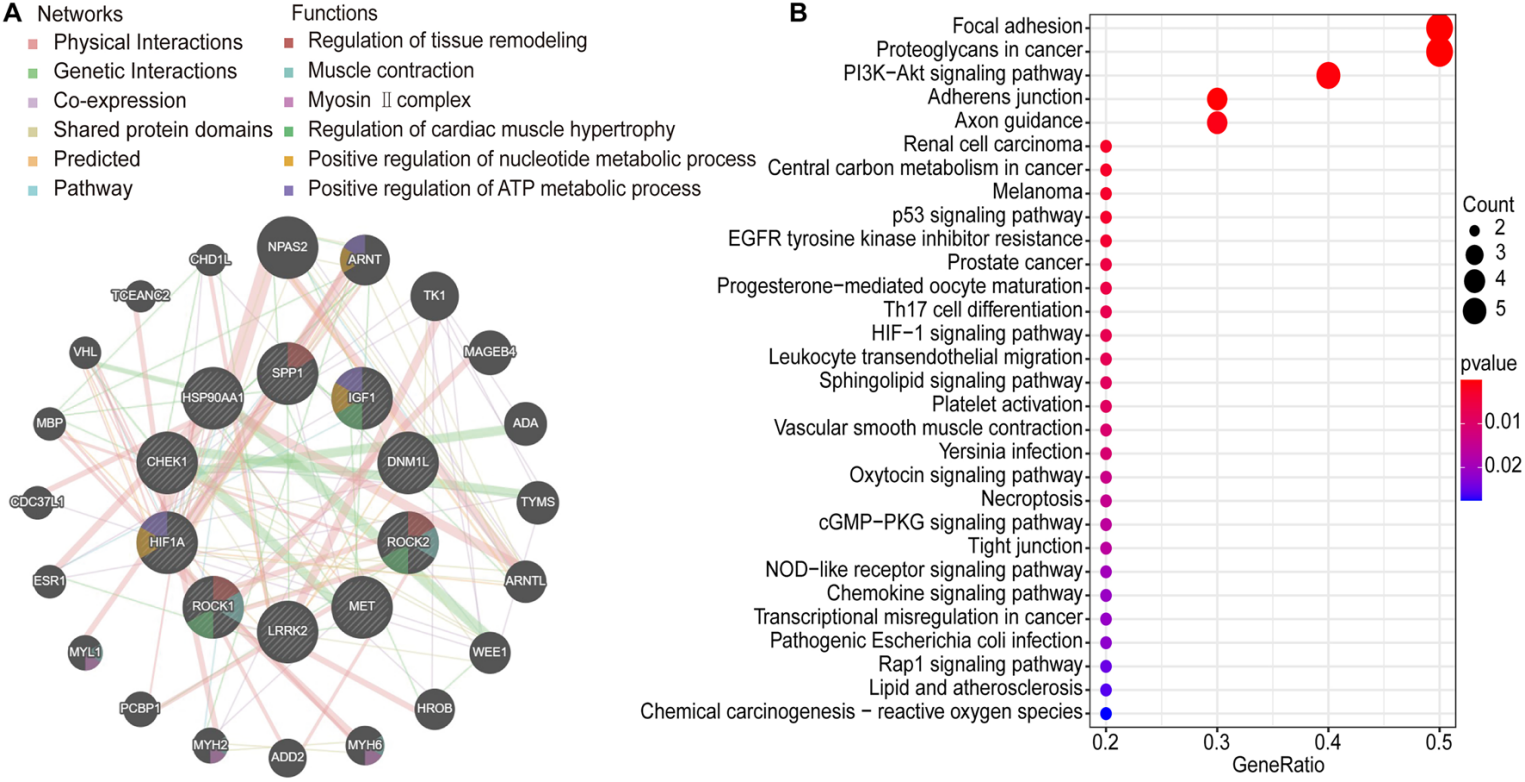
Functional analysis of Hub genes. (**A**) Gene–gene interaction network identification of Hub genes. (**B**) The top 10 Hub genes were enriched in the KEGG pathway analysis with *P* < 0.05. Warmer colors indicated higher statistical significance.

To construct the gene–gene interaction network involving the 10 Hub genes, we employed the GeneMANIA online analytic tool. The network layout positioned the anticipated genes in the outer circle, while the Hub genes were placed in the inner circle to facilitate visualization. The network analysis illustrated associations of the Hub genes with the regulation of tissue remodeling, muscle contraction, myosin II complex, regulation of heart hypertrophy, promotion of nucleotide metabolic activities, and enhancement of ATP metabolic processes (Fig. 6A). Furthermore, a KEGG enrichment analysis was performed on the 10 Hub genes (Fig. 6B). This analysis revealed significant associations of the Hub genes with focal adhesion, proteoglycans in cancer, and the PI3K-Akt signaling pathway.

### OPN^fl/fl^-TAGLN-Cre alleviation of HPH under hypoxia

**Figure 7:**
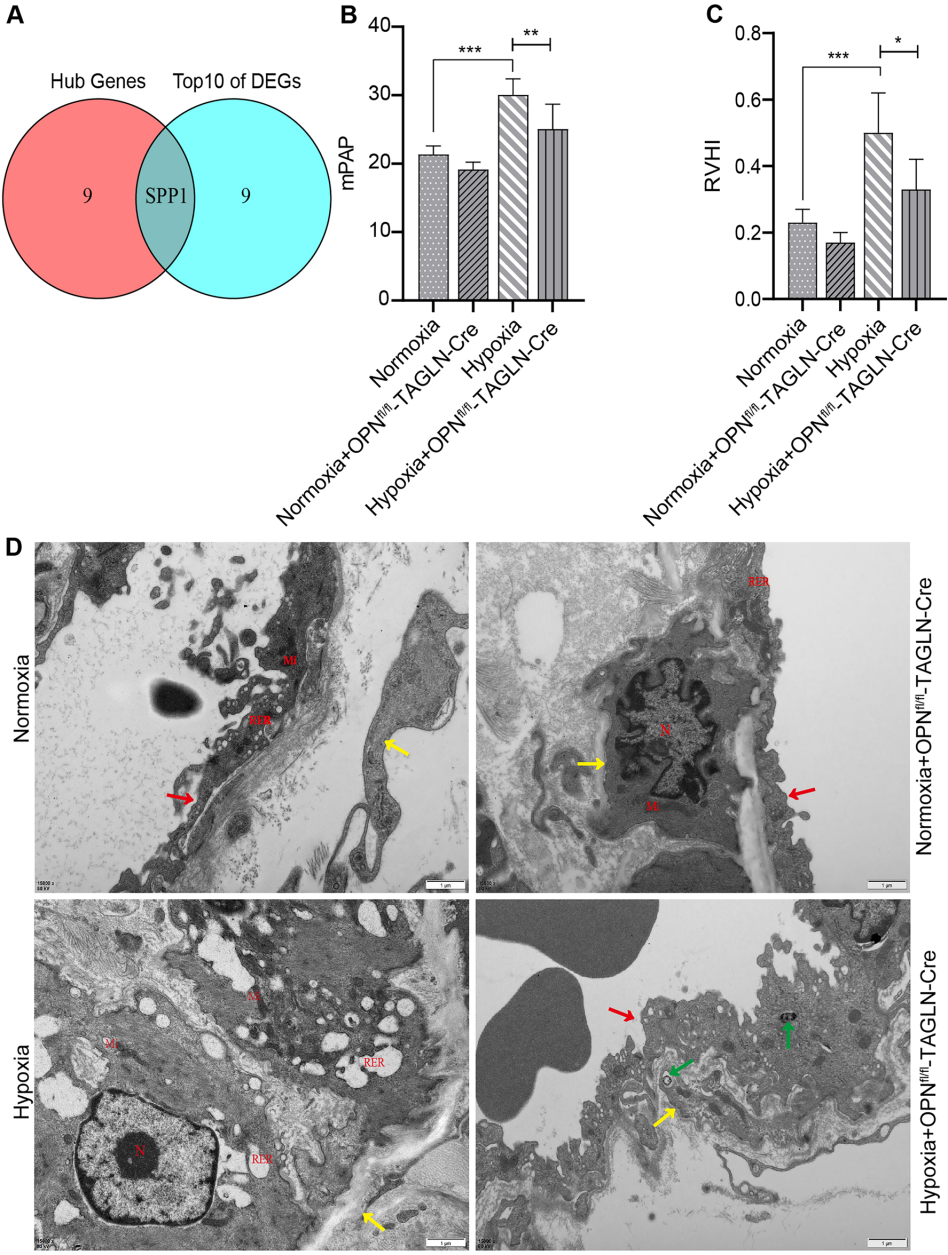
The effect of OPN knockdown in a hypoxic environment on RVHI, mPAP, and pathologic changes in the pulmonary arteries in HPH. (**A**) The intersecting gene between the top 10 DEGs and the hub genes was SPP1. (**B**) mPAP measurement. (**C**) RVHI measurement. (**D**) Sections of an electron microscope (scale bar: 1 µm) revealed altered pulmonary artery vascular cells. Mi (mitochondrion), N (nucleus), RER (rough endoplasmic reticulum). Pulmonary artery endothelial cells (red up arrow), pulmonary artery smooth muscle cells (yellow up arrow), autophagy (green up arrow). Results are representative of 7 independent experiments. Asterisks indicate statistically significant differences. *p < 0.05, **p < 0.01, ***p < 0.001.

Bioinformatics analysis revealed the significance of the SPP1 gene, also known as OPN, as an autophagy gene with a pivotal role in PAH. Identified as one of the top 10 genes among DEGs, OPN was further identified as a hub gene (Fig. 7A). Consequently, it was recognized as a gene of interest for subsequent investigations. The effects of OPN on hypoxic HPH were explored using gene-specific knockout technology. Our investigation yielded noteworthy results regarding mPAP (Fig. 7B) and RVHI (Fig. 7C). Notably, the hypoxia-exposed group exhibited a statistically significant increase in these parameters compared to the control group. Interestingly, the group subjected to hypoxia and lacking the OPN gene (OPN^fl/fl^-TAGLN-Cre) demonstrated reduced mPAP and RVHI values compared to the hypoxia-exposed group. Furthermore, pulmonary arteries in lung tissue were analyzed using transmission electron microscopy (Fig. 7D). The results revealed noticeable smooth muscle cells (SMC) abnormalities in the hypoxia-exposed group, contrasting with the normal appearance of SMC in the normoxia group. Notably, mice with OPN^fl/fl^-TAGLN-Cre exhibited increased autophagy in response to hypoxia, while SMC did not undergo anomaly. These findings suggest that downregulating OPN expression under low oxygen conditions mitigated the effects of HPH.

### OPN activation of PI3K inhibits the autophagy genes LC3B and Beclin1

**Figure 8:**
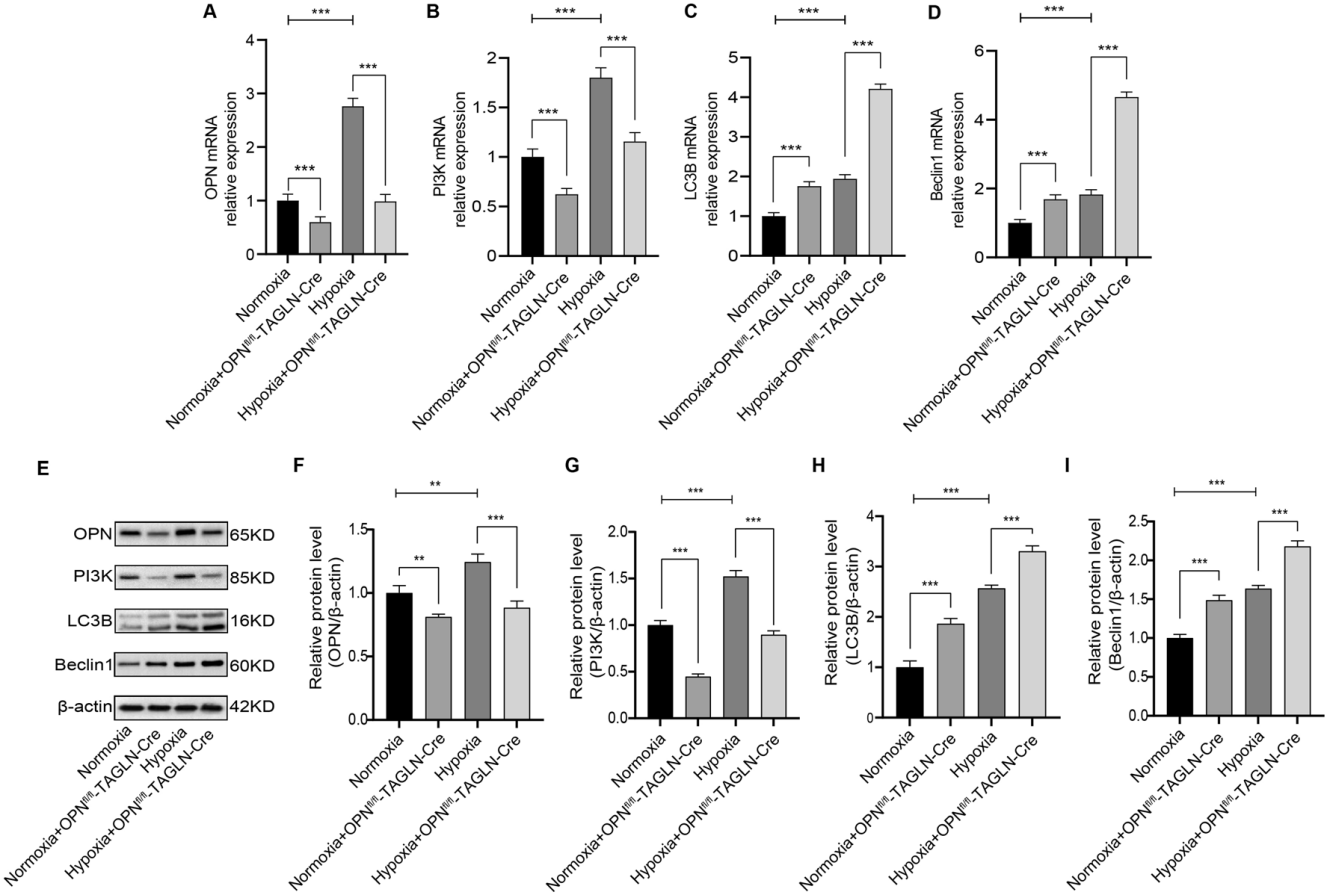
Inhibition of OPN in mouse lung tissue affected the expression of PI3K, LC3B, and Beclin1. (**A**) Relative OPN mRNA expression in lung tissues. (**B**) Relative PI3K mRNA expression in lung tissues. (**C**) Relative LC3B mRNA expression in lung tissues. (**D**) Relative Beclin1 mRNA expression in lung tissues. (**E**) OPN, PI3K, LC3B, and Beclin1 were analyzed by WB in the normoxia, normoxia + OPN^fl/fl^-TAGLN-Cre, hypoxia, and hypoxia + OPN^fl/fl^-TAGLN-Cre groups. (**F**–**I**) OPN, PI3K, LC3B, and Beclin1 proteins relative expression in the indicated groups. Results are representative of 3 independent experiments. Asterisks indicated statistically significant differences. **p < 0.01, ***p < 0.001.

The primary pathway associated with hub genes, as determined by KEGG analysis, was the PI3K-AKT signaling pathway. In the hypoxic environment of the animal model, it became evident that OPN influenced the levels of PI3K and autophagy. There is compelling empirical evidence supporting the idea that OPN plays a crucial role in regulating the PI3K signaling pathway and autophagic processes, ultimately affecting the invasive capability and functional behavior of cells^30,31^. To investigate this further, levels of OPN, PI3K, and autophagy-related genes and proteins such as LC3B and Beclin1 were measured in lung tissues collected from a mouse model of HPH. RT-PCR analysis revealed that OPN^fl/fl^-TAGLN-Cre mice exhibited significantly reduced expression levels of OPN and PI3K under both hypoxia and normoxic conditions (Fig. 8A,B), compared to the normoxia and hypoxia groups. Conversely, mRNA levels of LC3B and Beclin1 were increased in OPN^fl/fl^-TAGLN-Cre mice (Fig. 8C,D). WB analysis demonstrated that hypoxia stimulated increased expression of OPN, PI3K, LC3B, and Beclin1 compared to the group exposed to normal oxygen levels (Fig. 8E). Conversely, OPN^fl/fl^-TAGLN-Cre mice exhibited decreased levels of OPN and PI3K in both normoxic and hypoxic conditions, while simultaneously showing increased expression of autophagy proteins LC3B and Beclin1 (Fig. 8F–I). These findings suggest that increased OPN expression under low oxygen conditions contributes to the activation of the PI3K signaling pathway. Conversely, suppressing OPN expression enhances the production of autophagy genes and proteins.

### Involvement of OPN in PI3K-AKT signaling pathway affects autophagy in hypoxic PASMCs

**Figure 9:**
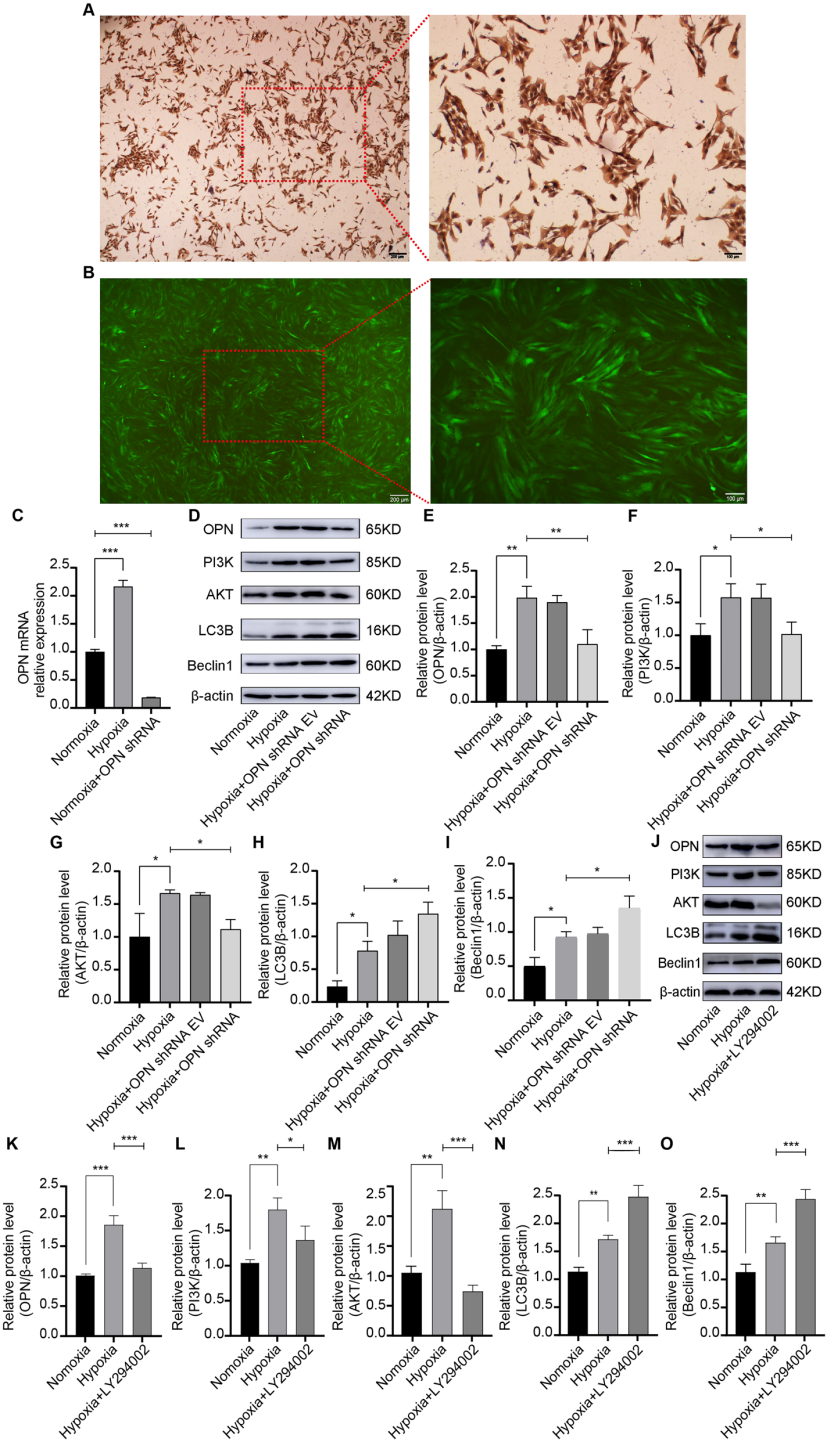
Lentiviral intervention utilizing OPN and the application of PI3K inhibitor augmented the expression of autophagy proteins by PASMCs under hypoxic conditions. (**A**) Immunohistochemistry results showed the identification of PASMCs; brown cells were PASMCs expressing α-SMA (scale bar: 200 μm/100 μm). (**B**) The green fluorescence indicated lentivirus that had entered PASMCs (scale bar: 200 μm/100 μm). (**C**) OPN expression under normoxia, hypoxia, normoxia + OPN shRNA conditions. (**D**) OPN, PI3K, AKT, LC3B, and Beclin1 were analyzed by WB in the normoxia, hypoxia, hypoxia OPN shRNA empty virus, and hypoxia OPN shRNA groups. (**E**–**I**) Protein expression levels for OPN, PI3K, AKT, LC3B, and Beclin1 were shown. (**J**) OPN, PI3K, AKT, LC3B, and Beclin1 were analyzed by WB in normoxia, hypoxia, and hypoxia + PI3K inhibitor (LY294002) groups. (**K**–**O**) OPN, PI3K, AKT, LC3B, and Beclin1 proteins relative expression in the indicated groups. PASMCs in all groups were incubated in normoxic (5% CO_2_ and 20% O_2_) or hypoxic (5% CO_2_ and 1% O_2_) environments for 48 h. Results are representative of 3 independent experiments. Asterisks indicated statistically significant differences. *p < 0.05, **p < 0.01, ***p < 0.001.

Vascular remodeling plays a critical factor in the pathogenesis of HPH, with the proliferation and hypertrophy of PASMCs significantly contributing to this process^32^. To elucidate the involvement of OPN, PI3K-AKT, and autophagy in vitro, PASMCs were cultured (Fig. 9A) and exposed to hypoxic and normoxic conditions for 48 h. The RT-PCR results (Fig. 9C) demonstrated an upregulation of OPN expression in PASMCs under hypoxic conditions compared to normoxia. Subsequently, lentiviral vectors carrying OPN shRNA were employed to target OPN expression in PASMCs, as indicated by the manifestation of green fluorescence (Fig. 9B). Following lentiviral transduction, PASMCs were exposed to were exposed to hypoxia and cultured. WB analysis (Fig. 9D) revealed a reduction in OPN expression in rat PASMCs transduced with OPN shRNA under hypoxic conditions compared to the hypoxic control group (Fig. 9E). Further investigation into the PI3K and AKT proteins exhibited elevated expression levels under hypoxic environments compared to normoxia. However, in hypoxic PASMCs subjected to OPN shRNA intervention, a significant decrease in the expression of both PI3K and AKT was observed (Fig. 9F–G). Moreover, the levels of autophagy-related proteins, such as LC3B and Beclin1, were found to be elevated in the hypoxia group compared to the normoxia group. Notably, hypoxic PASMCs treated with OPN shRNA displayed a notable increase in the expression of both LC3B and Beclin1 proteins (Fig. 9H–I). To further confirm the regulatory role of the PI3K-AKT signaling pathway in autophagy, a PI3K inhibitor (LY294002) was introduced to hypoxic PASMCs, followed by WB analysis (Fig. 9J). Remarkably, the expression levels of OPN, PI3K, and AKT were reduced in the hypoxic group treated with the PI3K inhibitor compared with the hypoxic control group. Conversely, the expression levels of Beclin1 and LC3B were increased (Fig. 9K–O). These findings suggest that suppressing OPN expression in PASMCs under hypoxic conditions promotes the production of autophagy-related proteins through modulation of the PI3K-AKT signaling pathway.

### Localization of autophagy proteins and formation of autophagosomes in PASMCs

**Figure 10:**
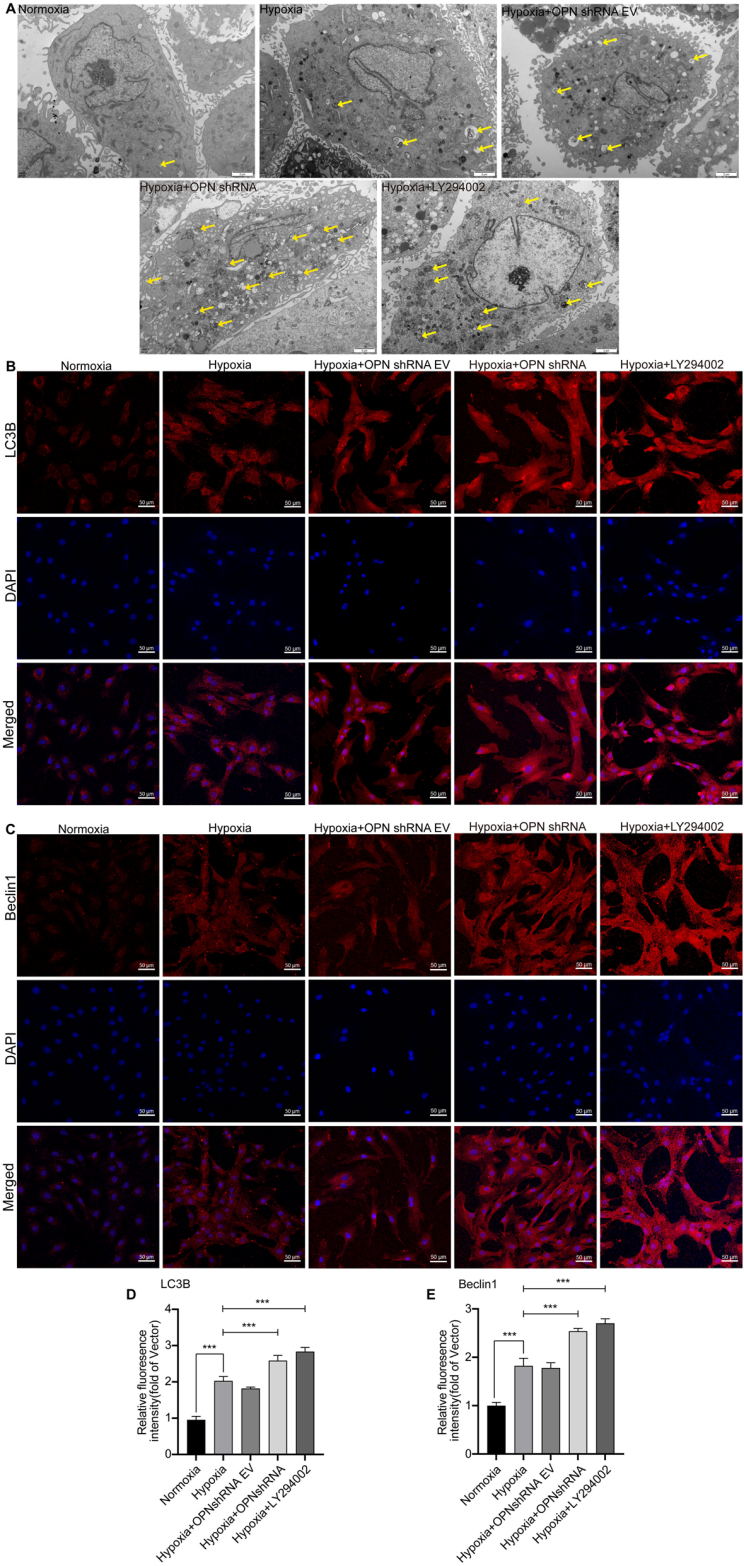
Quantitative expression of autophagosomes and autophagy-related proteins in PASMCs. (**A**) Electron microscopic analysis (scale bar: 2 μm) of the number of autophagosomes (yellow uparrow) in the indicated groups. (**B**,**C**) Immunofluorescence (scale bar: 50 μm) showed the fluorescence expression intensity of LC3B and Beclin1 in PASMCs. (**D**–**E**) Quantification of LC3B and Beclin1 immunofluorescence. PASMCs in all groups were incubated in normoxic (5% CO_2_ and 20% O_2_) or hypoxic (5% CO_2_ and 1% O_2_) environments for 48 h. Results are representative of 3 independent experiments. Asterisks indicated statistically significant differences. ***p < 0.001.

The association between OPN and autophagy has been well-documented in certain diseases^31,33^. However, the extent of autophagic activity in HPH needs to be further investigated. It is important to recognize that simply identifying autophagy proteins does not provide a comprehensive understanding of autophagy. Therefore, we conducted direct observations of autophagosomes within each experimental group (Fig. 10A). Notably, the hypoxic group exhibited a greater abundance of autophagosomes compared to the normoxic group. Importantly, the generation of autophagosomes in response to low oxygen levels was greater upon administration of OPN shRNA and LY294002, as opposed to hypoxia alone. To visually analyze the distribution of autophagy proteins within cells, we employed cellular immunofluorescence staining techniques to evaluate the presence of these proteins (Fig. 10B,C). Fluorescence microscopy revealed the cytoplasmic localization of LC3B and Beclin1 in PASMCs. Furthermore, under hypoxic conditions, the intensity of red fluorescence indicative of LC3B and Beclin1 expression was notably enhanced compared to normoxia. Interestingly, the combined treatment of hypoxia with hypoxia with OPN shRNA and LY294002 resulted in a further increase in red fluorescence intensity, specifically in LC3B and Beclin1 labeling (Fig. 10D,E). These observations underscored heightened autophagic activity in PASMCs during hypoxia, with suppression of OPN and PI3K leading to enhanced autophagy under hypoxic conditions.

### Inhibition of OPN and PI3K expression suppresses PASMCs proliferation under hypoxia

**Figure 11:**
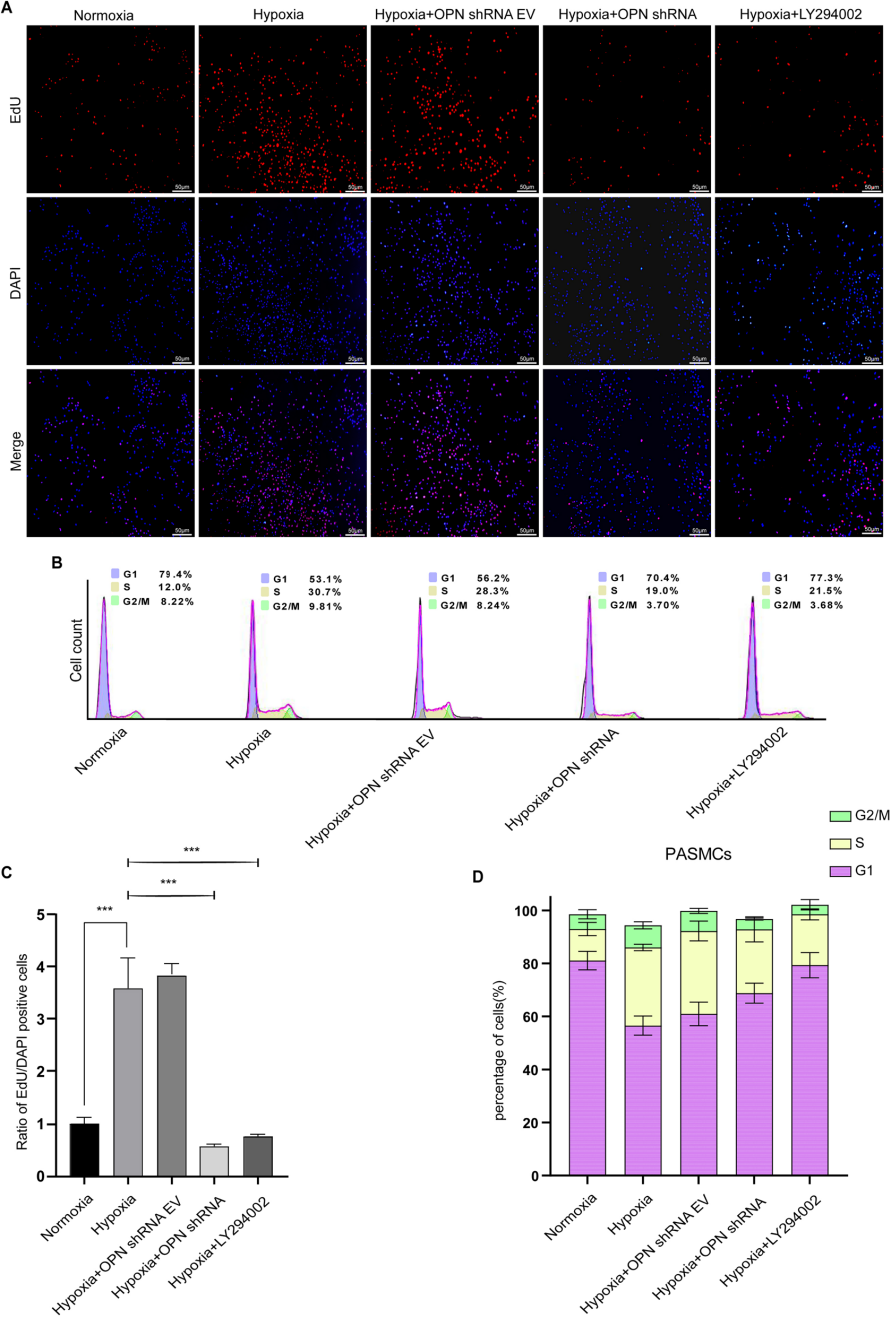
Under hypoxia, OPN and PI3K inhibition prevented PASMCs growth. (**A**) EdU staining of PASMCs showed alterations in cell proliferation (scale bar: 50 μm). (**B**) Flow cytometry analysis of the cell cycle. To calculate the percentage of cells in each phase, each set of cells was grown in the appropriate environment for 48 h before staining with PI. (**C**) Statistical chart showing the proportion of cells that were EdU-positive. (**D**) A plot of cell cycle dispersion distribution based on flow cytometry analysis. PASMCs in all groups were incubated in normoxic (5% CO_2_ and 20% O_2_) or hypoxic (5% CO_2_ and 1% O_2_) environments for 48 h. Results are representative of 3 independent experiments. Asterisks indicated statistically significant differences. ***p < 0.001.

Previous research has highlighted the rapid proliferation of PASMCs under hypoxic conditions^34^, emphasizing the importance of inhibiting their growth to ameliorate HPH. To investigate the proliferation status of PASMCs following increased autophagy, we conducted flow cytometry and EdU assays on each batch of cells. In the EdU assay, proliferating cells were identified as positive (Fig. 11A). Our findings demonstrated (Fig. 11C) that the hypoxia group exhibited a higher proliferation capacity compared to the normoxia group. However, treatment with OPN shRNA and the PI3K inhibitor LY294002 exhibited the potential to suppress PASMCs proliferation in a hypoxic environment. Additionally, flow cytometry analysis was conducted to investigate the cell cycle distribution. Under hypoxic conditions, there was a decrease in the number of cells in the G1 phase and an increase in the proportion of cells in the S phase and G2/M phase compared to normoxia (Fig. 11B,D). Conversely, hypoxic PASMCs treated with OPN shRNA and LY294002 displayed a higher proportion of cells in the G1-phase and a lower proportion of cells in the S-phase and G2/M-phase compared to the hypoxia group. These findings suggest that inhibiting OPN and PI3K expression in PASMCs under hypoxia effectively prevented PASMCs proliferation.

## Discussion

HPH involves various biological mechanisms, including proliferation, autophagy, and cell cycle alterations in PASMCs^35,36^. In this study, we observed that the suppression of OPN in vascular smooth muscle cells within a hypoxic mice model resulted in decreased mean mPAP and RVHI, ultimately ameliorating HPH. Furthermore, we discovered that OPN-regulated autophagy played a crucial role in modulating the proliferation of hypoxic PASMCs with the PI3K-AKT potentially serving as a key downstream signaling factor of OPN.

HPH, characterized by hypoxic pulmonary vasoconstriction leading to increased pulmonary vascular resistance and pulmonary artery pressure, is a key factor in the hypoxic proliferation of PASMCs. Previous studies have highlighted the occurrence of autophagy in PASMCs^37^. Therefore, elucidating the role of autophagy-related genes is important for developing interventions targeting HPH. By employing bioinformatics approaches, we identified candidate biomarkers of autophagy in PAH. DEGs in PAH were identified using the limma parameter method, followed by the analysis of DEGs, ARGs, and DMGs using WGCNA to select DEMARGs. Notably, among the top 10 DEMARGs identified as hub genes through CytoHubba, OPN emerged as a prominent gene, being not only upregulated but also serving as a hub gene. Moreover, KEGG analysis of hub genes revealed the PI3K-AKT signaling pathway as a key pathway implicated in HPH. However, the specific functions of OPN in HPH need to be further investigated. Elevated levels of PI3K have been associated with the development of various cardiovascular diseases, including PAH, atherosclerosis, and myocardial fibrosis^38–41^. and the inhibition of PI3K could inhibit the proliferation of blood vessels^42^ and promote apoptosis and autophagy^43,44^. Therefore, in this study, we focused on elucidating the link between OPN and PI3K in HPH.

OPN is an acidic arginine-glycine-aspartate adhesion glycoprotein^45^. OPN is primarily secreted by osteoblasts, osteoclasts, and hematopoietic cells^46^. However, recent studies have identified OPN expression in cells from various tissues, including PASMCs and vascular endothelial cells^47,48^. Under acute hypoxia conditions, vascular smooth muscle cells exhibit increased OPN expression, with elevated OPN levels correlating with increased autophagy^49^. OPN upregulation has been observed in pancreatic lung cancer cells, and knockdown of OPN leads to increased autophagic activity^50^. Autophagy, a process pivotal in controlling cell proliferation, has demonstrated inhibitory effects on lung cancer development when induced by exogenous Beclin1 supplementation. This augmentation of autophagy not only suppresses cancer cell growth but also mitigates angiogenesis and attenuates OPN expression^51^. Autophagy serves as a crucial regulator of fundamental cellular processes and significantly influences disease progression. Increased OPN expression coupled with autophagy inhibition in atrial fibrosis promotes the proliferative potential of fibroblasts, consequently exacerbating fibrosis^16^. Conversely, augmenting autophagy in PASMCs through pharmacological interventions has been shown to have protective effects, reducing their proliferative potential and potentially alleviating hypoxia-induced PAH^52^. Autophagy regulated by OPN exhibits a protective role in disease pathogenesis, suggesting that enhancing this process may represent a promising therapeutic strategy for treating HPH.

The PI3K pathway, situated downstream of OPN^53^, plays a pivotal role in regulating autophagy. OPN triggers the activation of the PI3K-AKT pathway in response to oxidative stress signals, primarily through integrin αVβ3^54^. The PI3K-AKT signaling pathway is crucial for fundamental cellular processes and exerts a significant impact on suppressing autophagy while stimulating proliferation^55,56^. Previous investigations have demonstrated the elevated concentration of PI3K in HPH compared to normal tissues, influencing cell division by modulating calcium levels within PASMCs^57^. Building upon this body of evidence, our hypothesis posits that the lack or inhibition of OPN in PASMCs could mitigate the progression of HPH by suppressing proliferation via PI3K-mediated autophagy.

To test the above hypotheses, we investigated differences in OPN expression between PAH and non-PAH. HPH is classified as a subtype of PAH, and to elucidate the role of OPN in HPH, mice with OPN deletion specifically in SMC were subjected to hypobaric oxygen chamber conditions to induce HPH. Our results demonstrated that OPN inhibition led to decreased RVHI and mPAP, accompanied by a significant enhancement in autophagy expression. This confirms the involvement of OPN in modulating autophagy expression. To validate the findings from pathway analysis, we conducted in vitro experiments. These experiments revealed that OPN knockdown reversed the effects of hypoxia on rat PASMCs proliferation and upregulated autophagy protein expression, providing further evidence of OPN’s influence on autophagy occurrence. Additionally, treatment with PI3K inhibitors on hypoxic PASMCs enhanced autophagy and inhibited cell proliferation, suggesting that OPN regulates autophagy via the PI3K-AKT pathway in PASMCs, with alterations in autophagy affecting proliferation. Based on these findings, we inferred that OPN regulates the PI3K-AKT pathway of autophagy, thereby influencing the thickening of the pulmonary artery smooth muscle layer in HPH.

This study has some limitations. Firstly, our approach for screening differential genes relied solely on one database, potentially limiting the comprehensiveness of our gene selection process. Additionally, during the screening phase, we did not prioritize the primary key pathway but instead opted for the PI3K-AKT pathway due to its relevance to autophagy. Furthermore, the validation of this pathway in vitro was compromised by the omission of a PI3K-AKT signaling pathway inhibitor in the normoxic PASMCs control group.

## Conclusions

In summary, our study demonstrates that OPN regulates the autophagy pathway via the PI3K-AKT signaling axis in PASMCs under hypoxic conditions. This augmentation of the protective autophagic response effectively prevents the remodeling of the pulmonary artery smooth muscle layer. To our knowledge, this is the first study to investigate the relationship between PI3K-AKT signaling regulated by OPN and autophagy in HPH. These findings provide a new research direction regarding the mechanisms of HPH and offer potential avenues for improving clinical diagnosis and developing targeted therapy for HPH.

### Supplementary Information

Supplementary Information.

## Data Availability

The dataset GSE113439 for this study can be found in the Gene Expression Omnibus database (https://www.ncbi.nlm.nih.gov/geo/query/acc.cgi?acc=GSE113439). The autophagy-related genes (ARGs) data for this study can be found in the Human Autophagy Database (http://hamdb.scbdd.com/home/index/). The gene–gene interaction network of Hub genes can be analyzed in an online web GeneMANIA (http://www.genemania.org).
