# Supplementary material for: OPN silencing reduces hypoxic pulmonary hypertension via PI3K-AKT-induced protective autophagy
Source: Sci Rep. 2024 Apr 15;14:8670. doi: 10.1038/s41598-024-59367-y (PMC11018812; doi:10.1038/s41598-024-59367-y)

**OPN silencing reduces hypoxic pulmonary hypertension via PI3K-AKT-induced protective autophagy**

**Rui Zhou**1**, Ran Li**2**, Qi Ding**3**, Yuwei Zhang**4**, Hui Yang**1**, Ying Han**1**, Chuanchuan Liu**5**, Jie Liu**1**, and Shenglan Wang**1,*

^1^ Qinghai University Medical Department, Xining, 810016, China

^2^ Zhengzhou Medical and Health Vocational College, Zhengzhou, 452385, China

^3^ Pathology Department of Tianjin Huanghe Hospital, Tianjin, 300110, China

^4^ Department of Public Health, School of Medical, Qinghai University, Xining, 810016, China

^5^ Key Laboratory of Hydatid disease, Qinghai University, Xining, 810001, China

^*^ Corresponding author: zlw6996@163.com

**Supplementary data**

**Table 1.** List of genes quantified by RealTime PCR. The table shows the primer pairs used in the amplification reaction

| Genes | Forward | Reverse |
| --- | --- | --- |
| OPN (mice)  OPN (rat) | AGCCACAAGTTTCACAGCCACAAGG  CCTGGCTGAATTCTGAGGGAC | CTGAGAAATGAGCAGTTAGTATTCCTGC  ATCAGTCACTTTCACCGGGAG |
| PI3K | TGAAGCAATGGGTGGAGCTCA | TGAGTCCTGATTCACACATAGCATCT |
| LC3B | CACAGTCTTTGTAAGGGCGGTTCT | GGCTTGCTTTAGTTGGAAGTGG |
| Beclin1 | AACTCTGGAGGTCTCGCTCT | TAGACCCCTCCATTCCTCAG |
| β-actin | CACTGTCGAGTCGCGTCC | TCATCCATGGCGAACTGGTG |


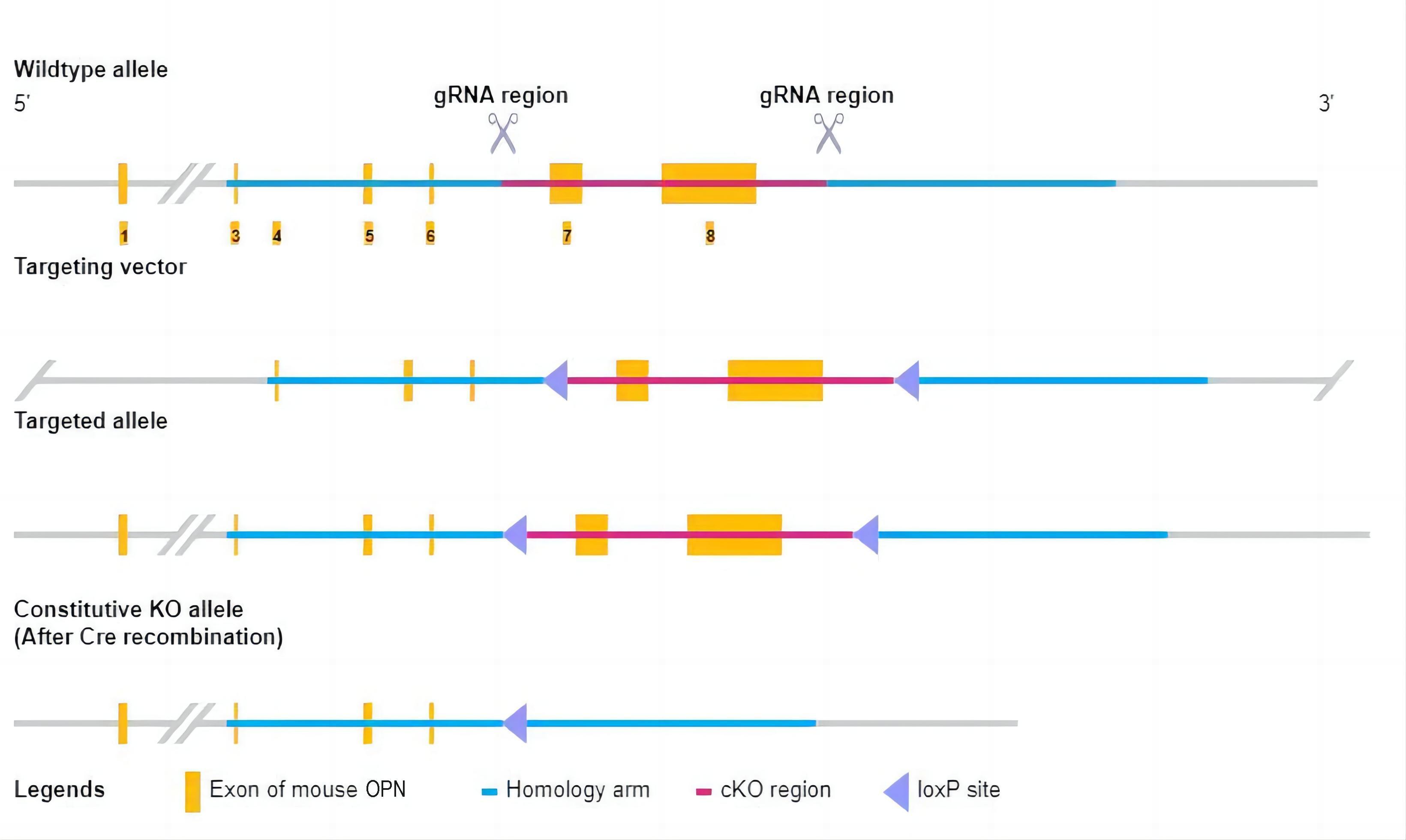


**FigureS1**: Flox mouse targeting construct.

| Genotyping | Flox | | Tagln |
| --- | --- | --- | --- |
| Flox/flox:  (Homozygotes) |  | 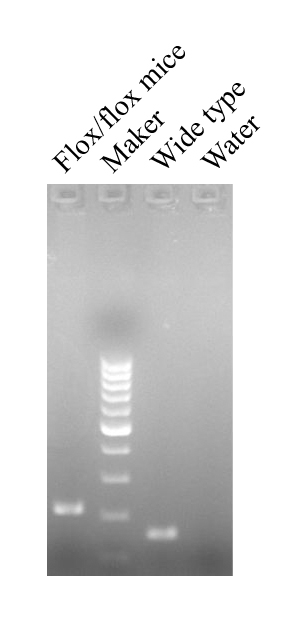 | 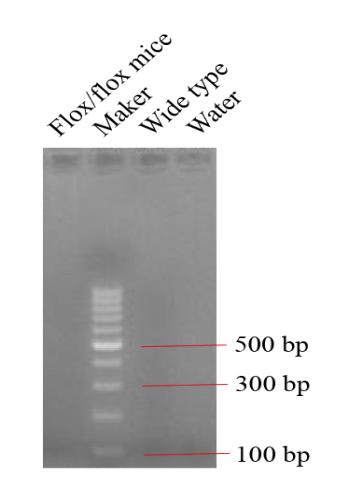 |
| Flox/flox-  TAGLN-Cre:  (Homozygotes) |  | 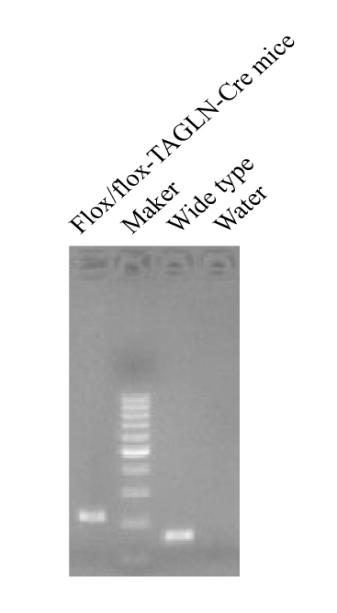 | 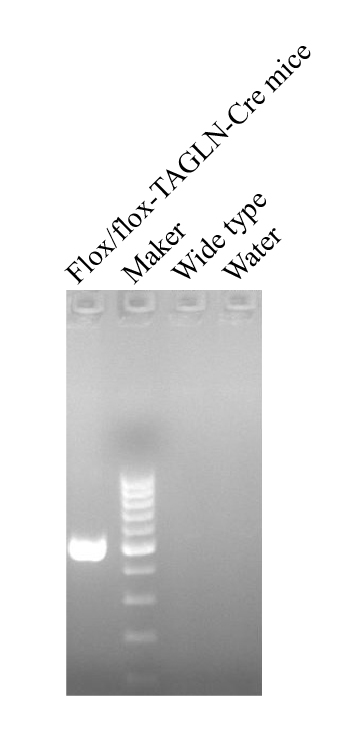 |

**FigureS2**: OPNflox/flox Pure Mice and OPNflox/flox-TAGLN-Cre Pure Mice.

Flox: F1: 5’-TCTAGTTCACTGTATGGATTTTGGC-3’

R1: 5’-CTGAAACATAGTTCCCTAAGACATCAG-3’

TAGLN: F1: 5’-CAGACACCGAAGCTACTCTCCTTCC-3’

R1: 5’-CGCATAACCAGTGAAACAGCATTGC-3’.

Homozygotes: one band with 218 bp. Heterozygotes: two bands with 218 bp and 153 bp. Wildty peallele: one band with 153 bp.

**Supplementary data**

**Original blots**

**
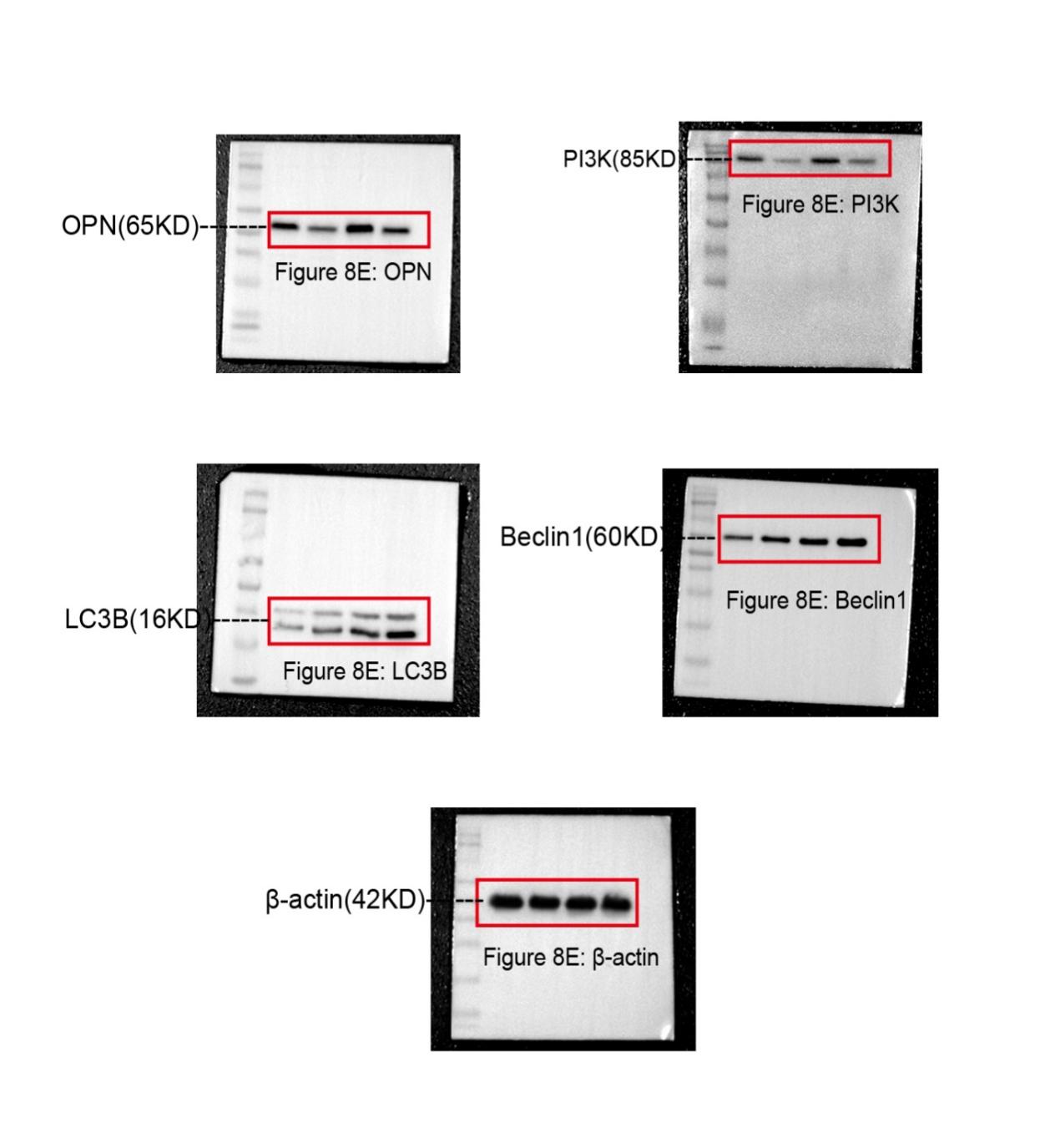
Figure 8E:**

**Figure9D:**

**
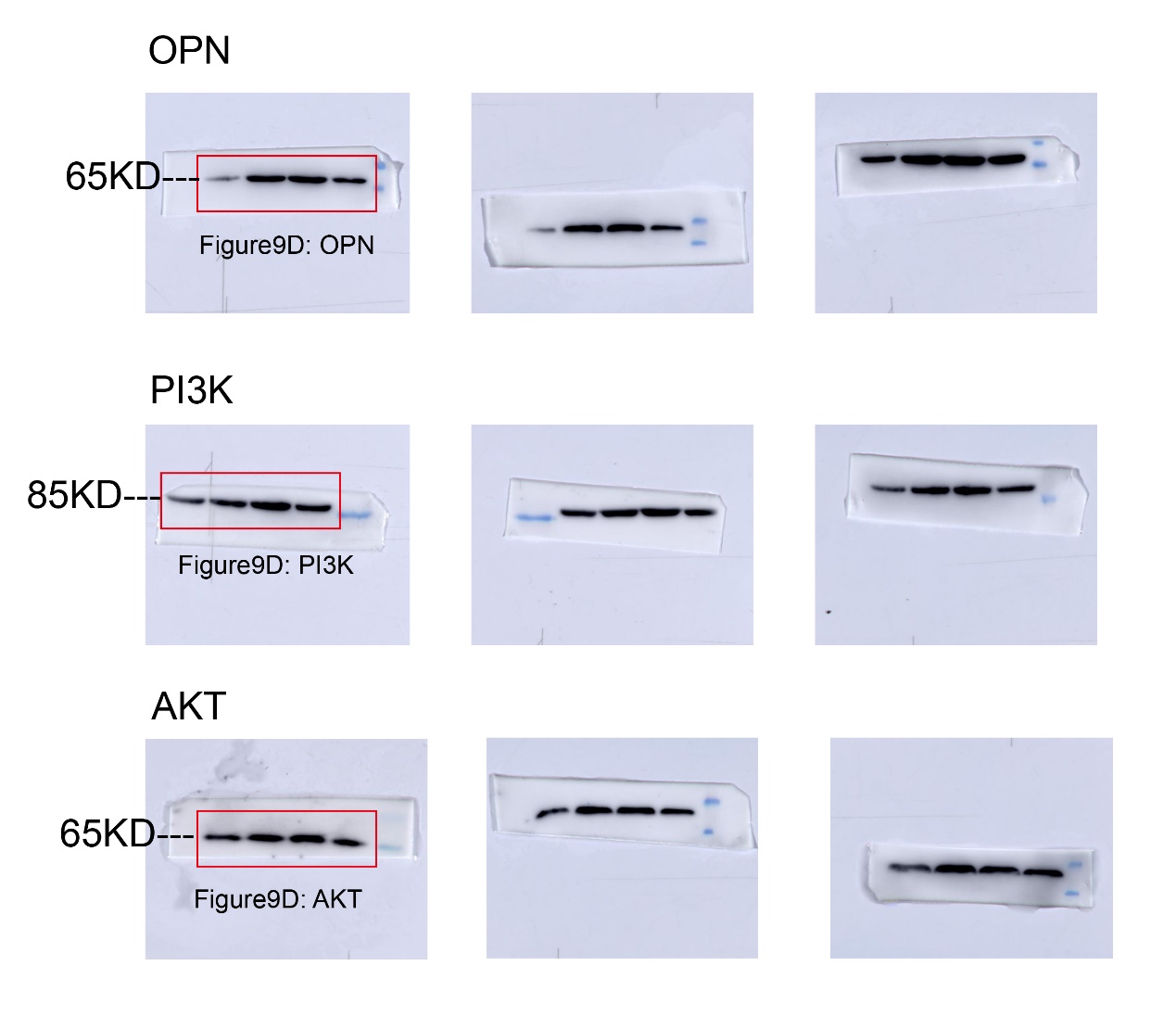
**


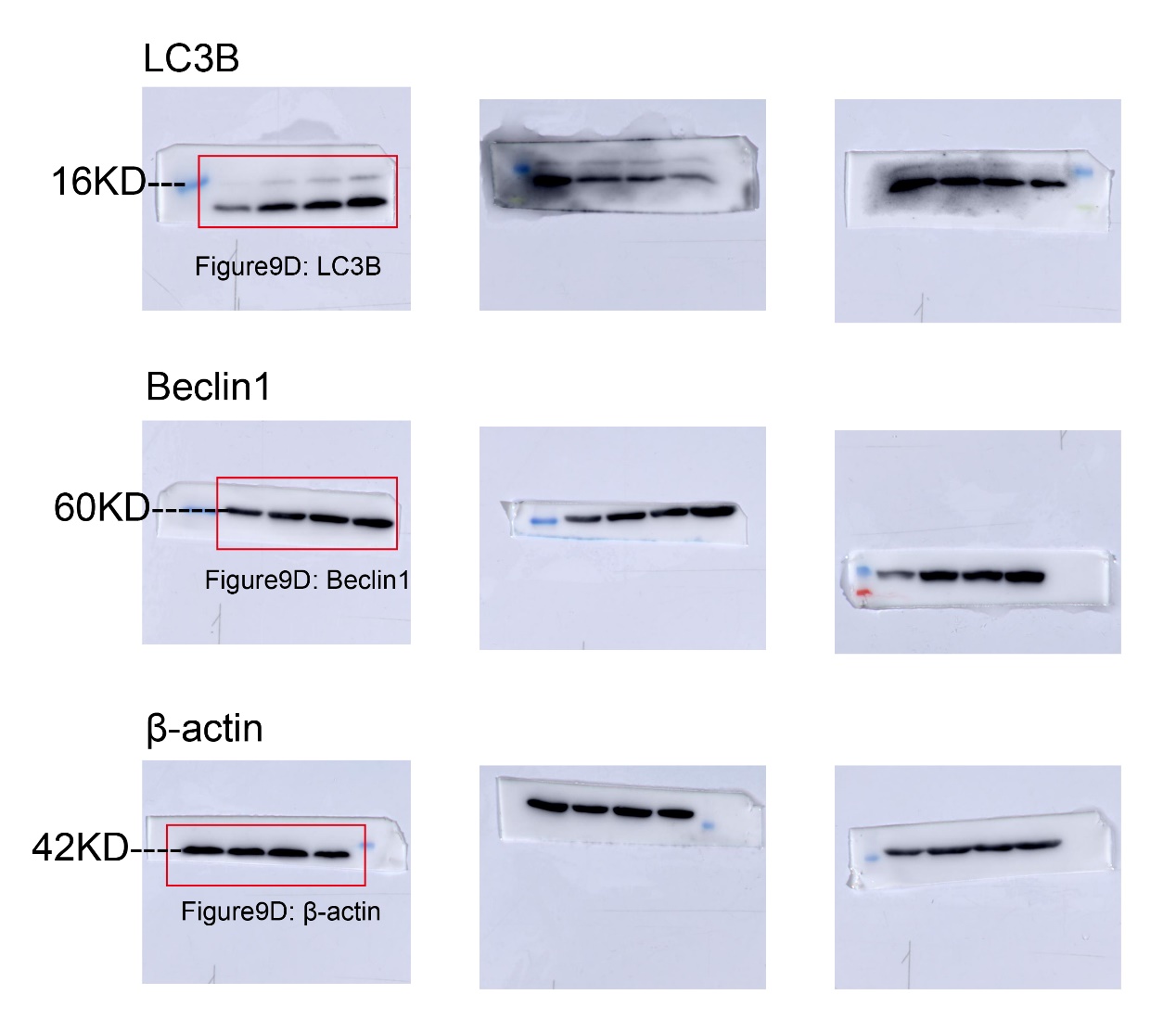


**Figure9J:**


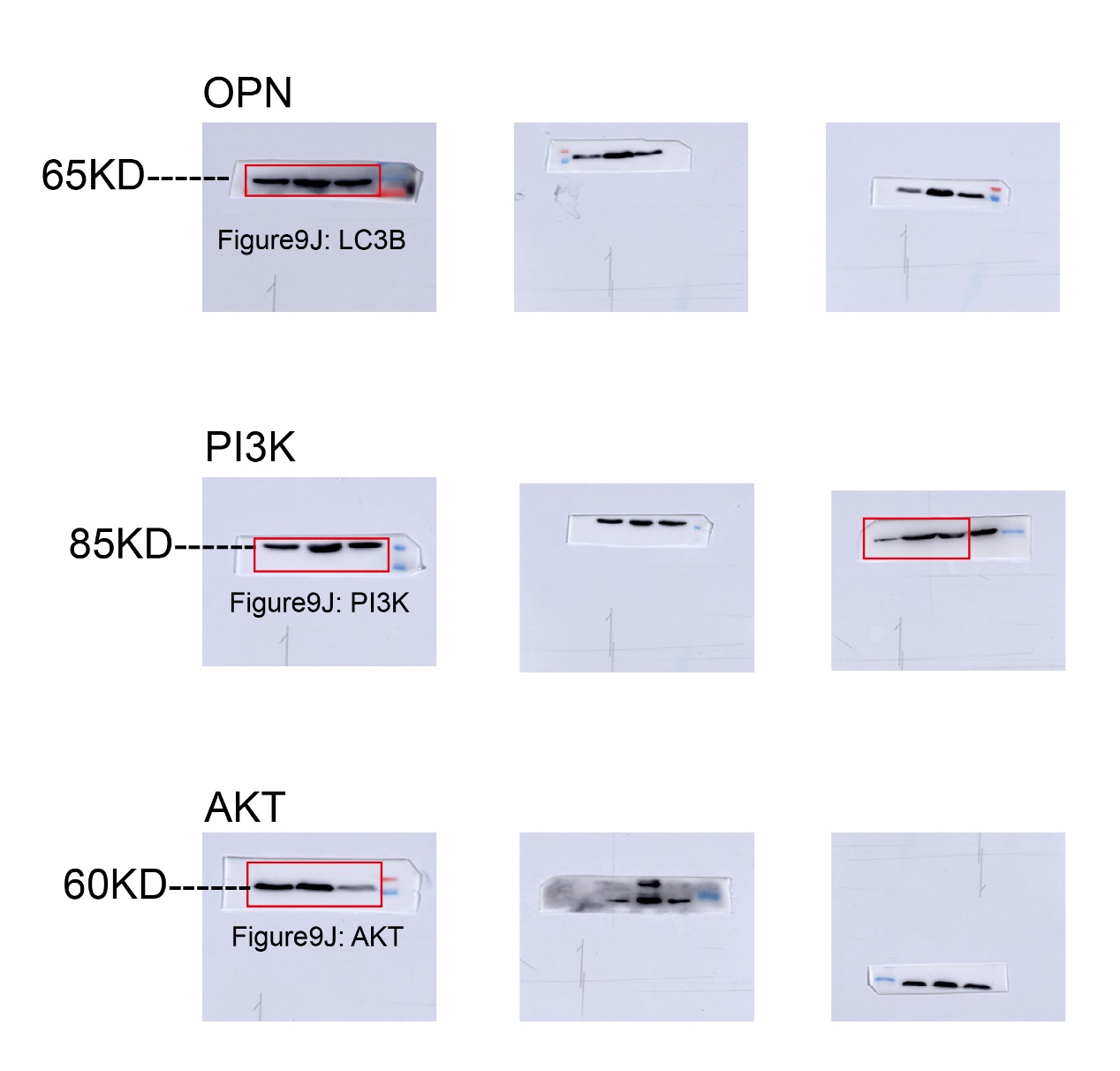


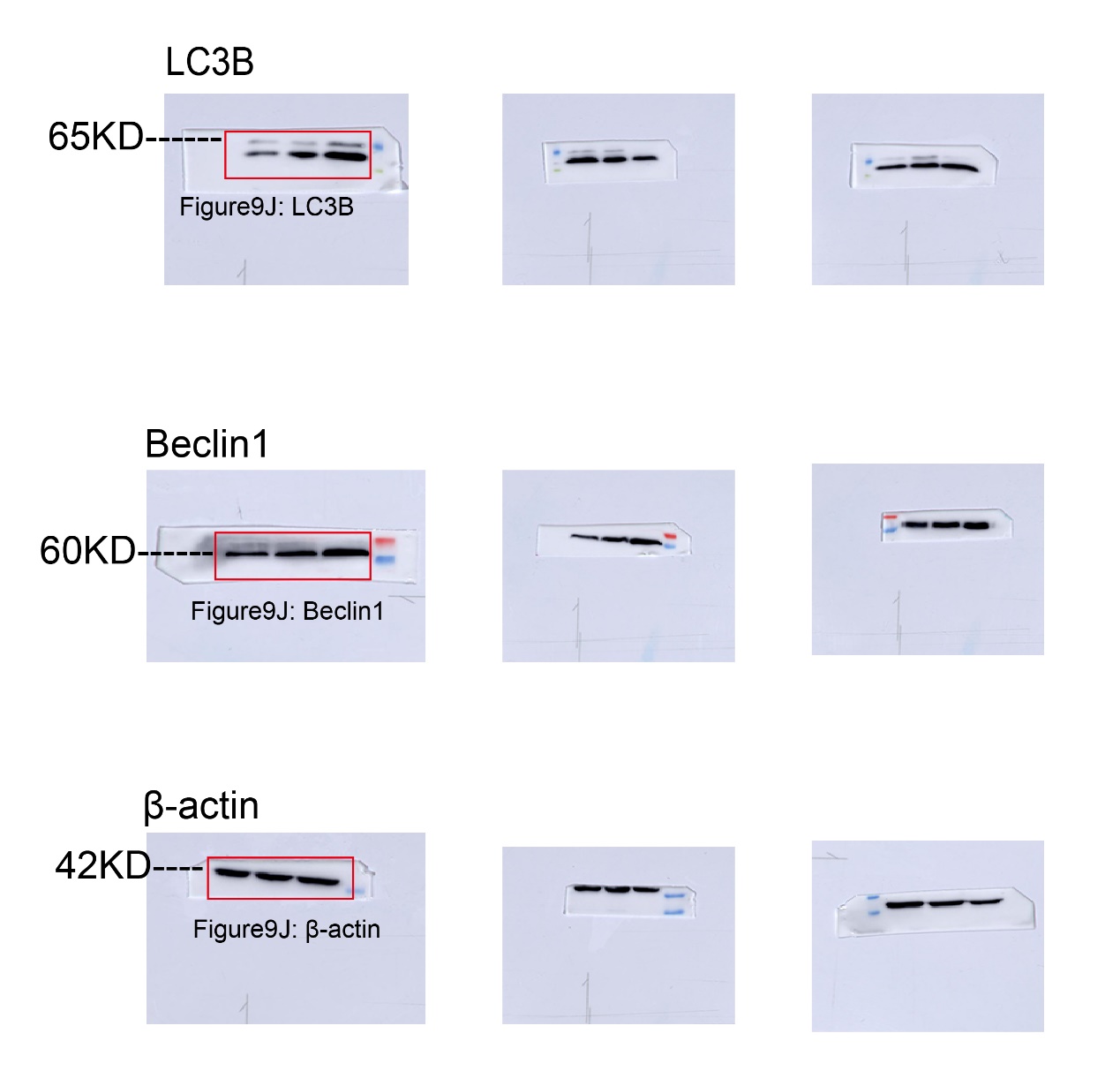

Supplement: Supplementary file 1 — Supplementary Information. [file 41598_2024_59367_MOESM1_ESM.docx]
